# Targeting the AHR–IDO1–kynurenine pathway in TREM2^+^ macrophages restores antitumor immunity in thyroid cancer

**DOI:** 10.3389/fimmu.2026.1826288

**Published:** 2026-07-21

**Authors:** Fei Le, Xianming Huang, Youfang Jiang, Saiping Lv, Yaoyao Chen

**Affiliations:** 1 Department of Head and Neck Tumor Surgery, Jiangxi Cancer Hospital, The Second Affiliated Hospital of Nanchang Medical College, Nanchang, Jiangxi, China; 2 Department of Medical Pathology, Jiangxi Cancer Hospital, The Second Affiliated Hospital of Nanchang Medical College, Nanchang, Jiangxi, China; 3 Department of Medical Laboratory, Jiangxi Cancer Hospital, The Second Affiliated Hospital of Nanchang Medical College, Nanchang, Jiangxi, China; 4 Medical College of Nanchang University, Nanchang, Jiangxi, China

**Keywords:** aryl hydrocarbon receptor-indoleamine 2,3-dioxygenase 1 pathway, immune evasion, kynurenine metabolism, single-cell omics, thyroid cancer, TREM2^+^ macrophages

## Abstract

**Introduction:**

Advances in single-cell technologies have provided detailed insight into cellular heterogeneity within tumor microenvironments. However, the regulatory mechanisms driving macrophage-mediated immune suppression in thyroid cancer remain incompletely understood.

**Methods:**

We applied integrated single-cell multi-omics approaches to tumor samples from patients and murine thyroid cancer models, including single-cell RNA sequencing, single-cell ATAC sequencing, mass cytometry, spatial/histological validation, and in vitro and in vivo functional assays.

**Results:**

We identified a prominent enrichment of TREM2^+^ tumor-associated macrophages within the thyroid cancer immune microenvironment. These macrophages displayed an immunosuppressive transcriptional and metabolic profile and promoted immune evasion through activation of the AHR–IDO1–kynurenine signaling axis, leading to impaired CD8^+^ T-cell function. Pharmacological inhibition of AHR and IDO1 reversed immune suppression and reduced tumor burden in experimental models.

**Discussion:**

These findings reveal a TREM2^+^ macrophage-driven immunometabolic mechanism in thyroid cancer and suggest that targeting the AHR–IDO1–kynurenine pathway may provide a potential strategy for improving antitumor immunity.

## Graphical Abstract

**Figure:**
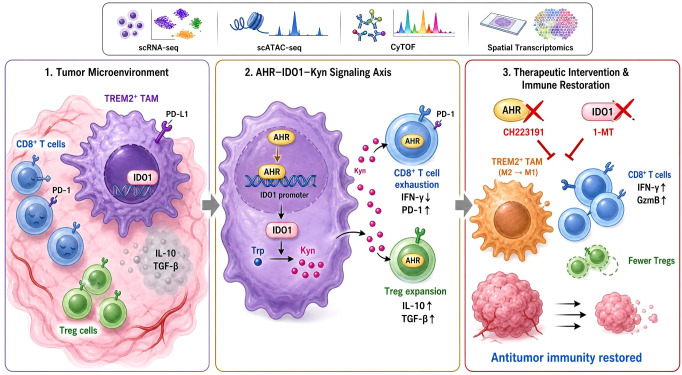
Integrated Multi-omics analysis of the molecular and metabolic mechanisms of TREM2+ macrophages in the TC immune microenvironment.

## Introduction

Thyroid cancer (TC) is the most common endocrine malignancy in clinical practice, and its global incidence continues to rise (1). Among all TC subtypes, papillary thyroid carcinoma (PTC) represents most cases (2). Although standard treatments, including surgical resection, radioactive iodine therapy, and hormone replacement, have generally produced favorable outcomes in PTC patients, their efficacy is still limited in high-risk cases characterized by invasiveness, distant metastasis, or recurrence (3). recently, immune checkpoint inhibitors (ICIs) have emerged as a breakthrough in cancer immunotherapy and have been widely applied across various malignancies (4, 5). However, numerous clinical studies have shown that the overall response rate to ICIs in TC remains low, suggesting the presence of a distinct immune tolerance mechanism within the tumor microenvironment that hampers therapeutic efficacy (6). As a result, elucidating the molecular mechanisms driving immune evasion in TC and innovatively optimizing related treatment strategies has become a critical area of research.

Tumor-associated macrophages (TAMs) are essential components of the tumor immune microenvironment and exhibit strong immunosuppressive activity during cancer progression (7). Prior studies indicate that TAMs promote tumor growth and metastasis through secreting immunosuppressive factors, stromal remodeling, and regulation of adaptive immune responses (8). In solid tumors, a TAM subpopulation characterized by TREM2 expression has attracted considerable attention. These TREM2^+^ macrophages have pronounced immunosuppressive functions and are tightly linked to poor prognosis and resistance to immunotherapy (9, 10). However, the metabolic context of this population differs across tumor microenvironments; for example, TREM2^+^ macrophages in hepatocellular carcinoma primarily exhibit lipid metabolic abnormalities, whereas those in gastric cancer rely on specific chemokine-axis programs (11, 12). At present, the single-cell transcriptional landscape, spatial distribution, and distinctive metabolic programming of TREM2^+^ macrophages in thyroid cancer remain insufficiently characterized. Therefore, determining whether these cells mediate thyroid-cancer-specific mechanisms of immune evasion is critical for evaluating their value as therapeutic targets.

Metabolic signaling pathways are tightly linked to the regulation of the tumor immune microenvironment, and among these, the aryl hydrocarbon receptor (AHR)-indoleamine 2,3-dioxygenase 1 (IDO1)-kynurenine (Kyn) metabolic axis has received growing attention in recent studies (13, 14). The AHR is a ligand-dependent transcription factor that senses metabolic cues in the microenvironment and activates the expression of genes involved in immunosuppression (15, 16). IDO1, a key enzyme in the tryptophan metabolism pathway, promotes the accumulation of Kyn when its activity is elevated. This, in turn, facilitates immune evasion by suppressing CD8+ T cell function and enhancing the expansion of regulatory T cells (Treg cells) (17, 18). Although the immunomodulatory function of this pathway has been partially characterized in certain solid tumors (19), its activation pattern and functional relevance in TC remain unclear. In particular, the potential interaction between this pathway and TREM2+ macrophages requires systematic investigation to provide mechanistic insights into the basis of immune tolerance in TC.

recently, advances in single-cell technologies have revolutionized the study of the tumor immune microenvironment. Cytometry by time-of-flight (CyTOF) enables high-throughput and quantitative analysis of multiple protein markers at the single-cell level, allowing for precise classification of immune cell subsets and their distinct phenotypes (20, 21). Additionally, single-cell assay for transposase-accessible chromatin using sequencing (scATAC-seq) has expanded our capacity to study transcription factor binding and associated regulatory networks with cellular specificity (22). In the field of tumor immunology, integrating these two technologies offers a comprehensive strategy to dissect functionally significant cell populations and uncover key signaling pathways relevant to cancer immunity. Here, we applied CyTOF and scATAC-seq for the first time to investigate the functions and molecular mechanisms of TREM2+ macrophages in the immune microenvironment of TC, thereby opening new avenues for therapeutic research.

Against this background, this study was designed to systematically elucidate the molecular mechanism by which TREM2^+^ macrophages drive immune tolerance in TC through the AHR-IDO1-Kyn signaling axis. By employing single-cell multi-omics tools, we generated a comprehensive profile of the immune phenotypes and metabolic characteristics of TREM2^+^ macrophages, and *in vitro* experiments then confirmed the role of this key signaling pathway in mediating TAM immunosuppression. The syngeneic BrafV600E transplantation model used in this study recapitulates the most common driver mutation in human PTC (BRAF V600E, 60%-90%) (23) and closely resembles clinical disease in histopathology, immune microenvironment remodeling, and targeted-therapy response (24), providing a reliable experimental platform for mechanistic and intervention studies. This work provides new theoretical insights into TC immune tolerance and explores the potential clinical value of targeting TREM2^+^ macrophages and metabolic signaling pathways, especially as a metabolic-immunologic intervention strategy for patients with poor responses to ICIs.

## Materials and methods

### Data sources and preprocessing

This study analyzed mouse thyroid cancer samples using publicly available single-cell transcriptomic databases. Tumor group data were derived from GSE232237 (https://www.ncbi.nlm.nih.gov/geo/query/acc.cgi), which includes single-cell RNA sequencing results from tumor tissues of a murine thyroid cancer model (n = 6). Control group data were downloaded from GSE182416, containing single-cell RNA sequencing results from normal mouse thyroid tissues (n = 3). Both datasets were generated using the 10x Genomics Chromium platform. Processed expression matrices and cell annotation files provided by GEO were downloaded and curated for downstream analyses, including single-cell clustering, differential expression analysis, and immune cell subpopulation validation. TCGA-THCA transcriptome data (tumor n = 511, normal n = 58) were downloaded from the GDC portal to compare expression differences of TREM2 and related immune regulatory genes between primary thyroid cancer tissues and normal thyroid tissues; expression values were log_2_(TPM + 1)-transformed for expression analysis.

Tumor tissues were collected from the murine thyroid cancer model and prepared as single-cell or single-nucleus suspensions by enzymatic digestion then mechanical dissociation. CD45^+^F4/80^+^ macrophages were isolated via flow cytometry and further sorted into TREM2^+^ and TREM2⁻ populations on the basis of TREM2 expression. The sorted cells underwent multi-omics analyses as follows: ① Bulk RNA-seq (n = 6; Illumina NovaSeq 6000, PE150, with a sequencing depth ≥ 20 million reads per sample); ② Single-cell ATAC-seq (n = 3; 10x Genomics Chromium Single Cell ATAC v1.1 library construction, sequenced on NovaSeq 6000); ③ Cell culture supernatants were collected at 12, 24, 36, and 48 hours (n = 6), then methanol precipitation under pre-chilled conditions. The internal standard [¹³C_6_]-kynurenine (final concentration: 50 ng/mL) was added, and kynurenine levels were quantified using ultra-high-performance liquid chromatography–tandem mass spectrometry (UHPLC-MS/MS) (C18 column, ESI positive ion mode, MRM 209→94); ④ Fresh-frozen tissue sections were prepared for spatial transcriptomics using the 10x Visium platform (n = 1, 10x Genomics, USA), along with IHC and IF validation with unified exposure settings and threshold parameters. All animals used in the present study were C57BL/6 mice bearing syngeneic Braf^V600E tumors. Tissue samples were harvested at the experimental endpoint, immediately cryopreserved, and processed for corresponding library preparation and downstream assays.

### scRNA-seq analysis

The single-cell expression matrix was processed using Seurat v4.3.0 (R 4.3.1). Cells were filtered according to the following criteria: fewer than 200 or more than 5,000 detected genes, total UMI counts exceeding 50,000 (potential doublets), or mitochondrial gene content greater than 20%. Data normalization was performed with the NormalizeData function (scale.factor = 1e4), and the top 2,000 highly variable genes were selected. Principal component analysis (PCA) was performed with 30 components retained, then UMAP visualization (n.neighbors = 30, min.dist = 0.3). Clustering was conducted with the Louvain algorithm with a resolution of 0.5. Cell-type annotation was performed with SingleR v2.4.1 with ImmGen and MouseRNAseq references, and then confirmed using canonical marker genes (e.g., Adgre1/F4/80, Itgam/CD11b, Pecam1, Cd3e, Ms4a1). TAMs were stratified into TREM2^+^ and TREM2- groups on the basis of Trem2 expression, using the upper quartile as the cutoff for “Trem2high.”

To further resolve TAM heterogeneity, cells annotated as TAMs were extracted and subjected to re-normalization, highly variable gene selection, PCA dimensionality reduction, and UMAP clustering. Differential marker genes for each TAM cluster were identified using Seurat’s FindAllMarkers function based on the Wilcoxon rank-sum test. According to marker-gene expression patterns, TAMs were further annotated into four subgroups: immunosuppressive TAMs (IS-TAMs), monocyte-like TAMs (Mono-TAMs), inflammatory TAMs (Inf-TAMs), and metabolic TAMs (Met-TAMs). The proportion of TREM2^+^ cells in each TAM subgroup was then calculated and extracted for visualization.

### Differential expression and functional analysis

Differential expression between TREM2^+^ and TREM2- TAMs was analyzed using Seurat’s FindMarkers function (Wilcoxon test, min.pct = 0.1, logfc.threshold = 0.25). Multiple testing correction was performed with the Benjamini-Hochberg method, with a false discovery rate (FDR) threshold of < 0.05. For bulk RNA-seq data derived from sorted macrophages, DESeq2 v1.38.3 (Bioconductor) was applied for normalization and differential expression analysis, applying |log2FC| ≥ 1 and FDR < 0.05 as thresholds. Results were visualized using volcano plots and violin plots. The volcano plots displayed log2FC on the x-axis and -log10(FDR) on the y-axis, with key genes (Ido1, Il10, Cd274) labeled for emphasis. Violin plots included overlaid scatter points to indicate the distribution of gene expression and the medians.

### Gene ontology and Kyoto encyclopedia of genes and genomes enrichment analysis

Differentially expressed gene (DEG) sets underwent enrichment analysis using clusterProfiler v4.8.1. GO enrichment was performed with the enrichGO function (OrgDb = org.Mm.eg.db v3.16.0, ont = “BP”), and KEGG pathway analysis was conducted with enrichKEGG (organism = “mmu”, database version updated to December 2023). An FDR < 0.05 was used as the significance threshold. Visualizations were generated with ggplot2 v3.4.4 and enrichplot v1.20.0, producing bar and bubble plots with enrichment ratios, enrichment scores, and -log10(q-values).

### Gene set enrichment analysis and single-sample gene set enrichment analysis module scoring

GSEA was carried out on the TAM expression matrix using GSEA v4.3.2 with gene sets from MSigDB v7.5.1 (Hallmark and KEGG). The parameters included 1,000 permutations (permutation type: gene_set) and an FDR q-value threshold of < 0.25. For AHR signaling, a combined gene set from MSigDB and published literature served to compute ssGSEA scores via GSVA v1.46.0 (method = “ssgsea”, tau = 0.25). Module activation scores were compared between the TREM2^+^ and TREM2- groups using the Wilcoxon test (FDR < 0.05). Results were visualized using density and boxplot overlays, along with receiver operating characteristic (ROC) curves generated by pROC v1.18.5.

### scATAC-seq analysis

Raw scATAC-seq data were aligned to the mm10/GRCm38 genome using Cell Ranger ATAC v2.1.0, and fragment files were generated for downstream processing. Subsequent analysis was conducted with ArchR v1.0.2. Cells with fewer than 3,000 unique fragments, transcription start site (TSS) enrichment scores below 7, or Blacklist Ratios exceeding 0.05 were removed. Dimensionality reduction was performed with latent semantic indexing (LSI), then UMAP visualization and clustering at a resolution of 0.5. The pipeline included addPeakMatrix, addMotifAnnotations(“cisbp”), and addPeak2GeneLinks. A ±200 kb window around the Ahr locus served to export coverage tracks, which were visualized in IGV v2.16 (Broad Institute) with a smoothing window size of k = 21. Global peak calling was conducted with MACS2 v2.2.9.1 with a significance threshold of q < 1e-5.

### Gene correlation analysis and visualization

In TREM2^+^ TAMs, correlations between AHR module scores and the expression of Il10 and Ido1 were calculated with two-tailed Spearman tests (α = 0.05). Scatter plots were generated with local polynomial regression (loess), with 95% confidence intervals (CIs). Additionally, expression data for AHR, IL10, and IDO1 were extracted from the TCGA-THCA cohort (GDC portal; FPKM transformed to TPM), and Pearson correlation analysis was carried out. Correlation coefficients and adjusted p-values were reported. Visualizations were created using ggpubr v0.6.0 and ComplexHeatmap v2.16.0.

### Spatial transcriptomics and histological validation

Spatial transcriptomic data were processed using the 10x Visium Spatial Gene Expression protocol. Alignment to the mm10/GRCm38 genome and generation of the spot-by-gene expression matrix and spatial coordinates were performed with Space Ranger v2.1.1. Data were imported into Seurat via SeuratData/SeuratDisk, then SCTransform normalization and batch correction (FindIntegrationAnchors/IntegrateData). Cell types were annotated on the basis of known marker genes or reference signatures. A gene module score representing the AHR-IDO1-Kyn axis was constructed. Regions in the top 10% of nCount_Spatial values were defined as “high transcriptional activity zones” and overlaid with expression maps for Trem2, Camk2a, and Nfatc1. Co-localization analysis was performed with Loupe Browser v6 and QuPath v0.4.4, with fixed thresholds used to calculate hotspot overlap ratios and Jaccard indices (**Supplementary Figure 1**).

### Establishment and grouping of the animal model

Under specific pathogen-free (SPF) barrier conditions (22 ± 2 °C, 50 ± 10% humidity, 12-hour light/dark cycle), syngeneic thyroid cancer models were established using 6–8-week-old C57BL/6 mice (n = 6 per group; purchased from Beijing Vital River Laboratory Animal Technology Co., Ltd.). The murine thyroid cancer cell line BVE (BRAF^V600E-induced tumor cell line), which carries the Braf^V600E mutation and was derived from spontaneous PTC tumors in thyroid-specific Braf^V600E knock-in transgenic mice, was used for tumor implantation; its genetic background is highly consistent with BRAF^V600E-mutant human PTC and had been verified by sequencing. Cells were suspended in phosphate-buffered saline (PBS; Ca²^+^/Mg²^+^-free; Gibco, 10010023) and Matrigel (Corning, 356234) at a 1:1 ratio and subcutaneously injected into the right dorsal/axillary region at 1 × 10^6^ cells in 100 μL. Injections were carried out under isotonic conditions while avoiding air bubbles. Starting from the day of inoculation, tumor dimensions were measured every 24 hours using an electronic caliper (Mitutoyo, 500-196-30), and tumor volume was calculated as V = (L × W²)/2, where L is the length and W is the width. When tumor volume reached approximately 100 mm³, mice were assigned to treatment groups by block randomization and treatment was initiated (defined as day 0). Baseline tumor volumes were compared across groups by one-way ANOVA to confirm balance after randomization. Animals designated for histological and multi-omics analyses were euthanized on day 28, and tissues were immediately collected following perfusion with pre-chilled sterile PBS, then either snap-frozen in liquid nitrogen or fixed at 4 °C.

Animal Anesthesia and Euthanasia:​ for all procedures that could cause discomfort (including tumor cell seeding), mice were anesthetized via inhalation of 3% isoflurane (RWD Life Science, China) in an induction chamber, and anesthesia was maintained with 1.5-2% isoflurane​ delivered via a nose cone. At the end of the experiment (day 28), animals were humanely euthanized. Euthanasia was carried out by intraperitoneal injection of an overdose of sodium pentobarbital​ (150 mg/kg; Sigma-Aldrich) then cervical dislocation to ensure death. All efforts were made to minimize animal suffering.

Group 1 (Adoptive Transfer of TREM2+ Macrophages): Control: An equal volume of buffer, intratumoral injection, using the same schedule as the TREM2+ group; TREM2-: 5 × 105 TREM2- macrophages per dose, intratumoral injection, once every 3 days, 4 doses over 14 days; TREM2+: 5 × 105 TREM2+ macrophages per dose, intratumoral injection, once every 3 days, 4 doses over 14 days.

Group 2 (AHR/IDO1 Inhibition and Combination Treatment): Control: An equal volume of normal saline, intraperitoneal (i.p.) injection, once every 48 hours for 14 days; AHR inhibitor: CH223191, 1 mg/kg, i.p., once every 48 hours for 14 days; IDO1 inhibitor: 1-methyl-D-tryptophan (1-MT), 50 mg/kg, i.p., once every 48 hours for 14 days Combination: CH223191 + 1-MT (doses and frequency as above), i.p., once every 48 hours for 14 days (**Supplementary Figure 2**).

### Mass cytometry (CyTOF)

On day 28, BrafV600E subcutaneous tumor tissues were harvested and digested at 4 °C for 30 minutes with Collagenase IV (1 mg/mL, Gibco, 17104019) and DNase I (50 U/mL, Roche, 10104159001). The cell suspension obtained was filtered through a 40 μm strainer (Falcon, 352340) and treated for red blood cell lysis (BioLegend, 420301). Cells were incubated with anti-mouse CD16/32 (Fc block; BioLegend, 101320) for 10 minutes, and cell viability was verified as ≥85% using trypan blue exclusion. Live/dead discrimination was performed with cisplatin (Fluidigm, 201064; room temperature, 5 minutes), then fixation with 1.6% paraformaldehyde (Electron Microscopy Sciences, 15710). The metal isotope-tagged antibody panel included markers for TREM2, AHR, IDO1, PD-L1, MHC-II, CD80, CD86, and core myeloid/lymphoid lineage antigens. Cells were incubated with the antibody cocktail at 4 °C for 30–60 minutes, washed with PBS containing 0.5% bovine serum albumin (BSA) (Sigma-Aldrich, A9647), and stained overnight at 4 °C with Ir191/193 DNA intercalator (Fluidigm, 201192B). Before acquisition, cells were mixed with EQ Four Element Calibration Beads (Fluidigm, 201078) and analyzed using the Helios mass cytometer (Fluidigm) at a flow rate of 300–500 events per second. Bead-based normalization was conducted with CyTOF Software v7. Debris and doublets were removed by gating on event length and Ir DNA signals, and dead cells were removed on the basis of cisplatin staining. Data were transformed using arcsinh(x/5). Within the CD45^+^F4/80^+^CD11b^+^ population, TREM2^+^ and TREM2- subpopulations were defined according to the TREM2 expression threshold. Positive rates and mean fluorescence intensity (MFI) values were exported. Cross-batch consistency was ensured using a standardized gating template and internal bead-based normalization.

### Multicolor flow cytometry

Tumors were harvested at the experimental endpoint and gently digested for 30 minutes using Collagenase IV, Elastase (1 U/mL; Worthington, LS006365), and DNase I. The cell suspension obtained was filtered through a 70 μm strainer, followed by red blood cell lysis. Dead cells were labeled with Fixable Viability Dye eFluor™ 780 (Thermo Fisher, 65-0865-14). The gating sequence was as follows: FSC/SSC to exclude debris and doublets → CD45^+^ total immune cells → myeloid cells (F4/80^+^CD11b^+^) and T cells (CD3^+^, further subdivided into CD8^+^/CD4^+^; Foxp3 intranuclear staining). The antibody panel included CD45-APC/Cy7 (BioLegend, 103116), CD3-PE/Cy7 (100320), CD8-FITC (100706), PD-1-BV421 (135218), F4/80-PerCP/Cy5.5 (123128), CD86-PE (105008), CD206-APC (141708), IFN-γ-APC (505810), Ki-67-PE (652404), and Foxp3-Alexa647 (126408); fluorescence-minus-one (FMO) controls served to guide gating thresholds. For intracellular cytokine detection, cells were stimulated with PMA (50 ng/mL; Sigma-Aldrich, Cat# P8139), ionomycin (1 μg/mL; Sigma-Aldrich, Cat# I0634), and Brefeldin A (5 μg/mL; BioLegend, Cat# 420601) for 4–5 hours prior to staining. Samples (≥1 × 10^5^ events per sample) were acquired using a BD LSRFortessa flow cytometer (BD Biosciences) and analyzed with FlowJo v10.9. A unified logical gating strategy was applied to all flow cytometry data. Intact cells were first selected on FSC-A/SSC-A plots to exclude debris, followed by FSC-A/FSC-H gating to remove doublets. Viable cells were selected using the Fixable Viability Dye eFluor™ 780-negative gate, and CD45^+^ cells were then gated to enrich total immune cells. For macrophage analysis, F4/80^+^CD11b^+^ macrophages were gated within the CD45^+^ population, and CD86 and CD206 expression was further analyzed. For T cell subset analysis, CD3^+^ T cells were gated within CD45^+^ cells and then separated into CD8^+^ and CD4^+^ subsets. Positive thresholds for intracellular cytokines (IFN-γ and Granzyme B) and Ki-67 were set using fluorescence-minus-one (FMO) controls. At least 1 × 10^5^ viable-cell events were acquired for each sample, and the representative gating strategy is provided in **Supplementary Material 1**. For the *in vitro* co-culture assay, macrophages were pretreated for 48 hours with the selective AHR inhibitor CH-223191 (10 μM) or an equal volume of vehicle control, then co-cultured with T cells. The same antibody panel served to evaluate changes in the proportion of Treg cells (CD4^+^CD25^+^Foxp3^+^).

### Hematoxylin and eosin staining

Tumor tissues were fixed in 10% neutral-buffered formalin, routinely paraffin-embedded, and serially sectioned at 4-6 μm. Sections were deparaffinized in xylene, rehydrated through a graded ethanol series, stained with hematoxylin for 5 minutes, differentiated with 1% acid alcohol, and rinsed in running water for bluing. Eosin staining was then performed for 2 minutes, followed by graded ethanol dehydration, xylene clearing, and mounting with neutral resin. All HE-stained slides were scanned using a digital pathology scanner (Pannoramic MIDI, 3DHISTECH) for histomorphological evaluation.

### Immunohistochemistry

Tumor tissues were fixed in 10% neutral-buffered formalin (Sigma-Aldrich, HT501128) for 24 hours, embedded in paraffin, and sectioned at 4-6 μm thickness. Sections were deparaffinized with xylene, rehydrated through a graded ethanol series, and treated with 3% H2O2 for 10 minutes to block endogenous peroxidase activity. Antigen retrieval was performed with citrate buffer (pH 6.0; Solarbio, P0081) at 95-98 °C for 15 minutes. Non-specific binding was blocked with 5% BSA (Solarbio, A8010) at room temperature for 30 minutes. Primary antibodies were incubated overnight at 4 °C: CD8 (CST, 85336, 1:200), Foxp3 (Abcam, ab20034, 1:200), and TREM2 (Abcam, ab209814, 1:200). Sections were then incubated with HRP-conjugated secondary antibody (ZSGB-BIO, PV-9001) at room temperature for 1 hour, developed with DAB substrate (ZSGB-BIO, ZLI-9018), and counterstained with hematoxylin (Servicebio, G1004). Slides were scanned using the NanoZoomer scanner (Hamamatsu, NanoZoomer-SQ C13140-01), and ≥5 random high-power fields per slide were quantitatively analyzed using QuPath v0.5.1 with unified thresholding and area-based filtering.

### TREM2-region segmentation and metabolite detection

Following immunostaining, whole-slide imaging was performed with a digital pathology scanner (Pannoramic MIDI, 3DHISTECH, Hungary). The TREM2 signal was digitally quantified using the HALO image analysis software (Indica Labs, USA). Based on positive pixel count thresholds, regions of TREM2high and TREM2low expression were defined within the same tumor section. Serial adjacent unstained sections were applied for macro-dissection (MMD), guided by previously annotated regions, to collect tissue samples from TREM2high and TREM2low areas, respectively. Samples were homogenized, and metabolites were extracted for quantification of Kyn levels using liquid chromatography-tandem mass spectrometry (LC-MS/MS; Q Exactive Plus, Thermo Fisher, USA). For correlation analysis, IDO1 activity and kynurenine concentration measurements were both derived from paired TREM2^high^ and TREM2^low^ region samples obtained from the same mouse tumor through serial sectioning and macro-dissection.

### ELISA

A commercial sandwich ELISA kit served to quantify IFN-γ, IL-10, TGF-β, and Arg1 (Thermo Fisher Scientific, USA; eBioscience, USA; R&D Systems, USA) according to the manufacturer’s instructions. Supernatants from co-culture assays were collected after 24-48 hours of incubation. Tumor tissues were homogenized, and total protein concentrations were determined using the BCA Protein Assay Kit (Thermo Fisher Scientific, USA) before sample dilution for analysis. Standard solutions and 100 μL of each sample were added to antibody-precoated wells of a microplate (Nunc MaxiSorp™, Thermo Fisher Scientific, USA) and incubated at room temperature for 1 hour, then five washes with PBS containing 0.05% Tween-20 (Sigma-Aldrich, USA). Biotinylated detection antibodies and horseradish peroxidase (HRP)-conjugated streptavidin (Sigma-Aldrich, USA) were sequentially added, with incubation periods of 1 hour and 30 minutes, respectively. After washing, TMB substrate (Solarbio, China) was added and allowed to react for 10–15 minutes. The reaction was stopped with 2N H2SO4 (Sinopharm Chemical Reagent, China), and absorbance was measured at 450 nm with a reference wavelength of 570 nm using a SpectraMax iD3 microplate reader (Molecular Devices, USA). A four-parameter logistic regression model served to fit the standard curves (R2 ≥ 0.99), and sample concentrations were calculated in pg/mL. For tissue samples, results were normalized to total protein content and expressed as pg/mg protein. Each sample was analyzed in at least two technical replicates, with a minimum of three biological replicates for *in vitro* experiments and n = 6 for *in vivo* experiments. The standard curves exhibited strong linearity (R2 ≥ 0.99), with an intra-assay coefficient of variation (CV) ≤ 15% and spike recovery rates between 80% and 120%.

### Western blot

Tumor tissues or cells were lysed using RIPA buffer (Beyotime, Cat# P0013B) supplemented with protease and phosphatase inhibitors (Beyotime, Cat# P1005). Lysates were centrifuged at 12,000 g for 10 minutes to remove debris. Protein concentration was quantified using a BCA assay (Thermo Fisher Scientific, Cat# 23227), and 20-40 μg of protein per well was loaded for SDS-PAGE separation, then transfer to PVDF membranes (Millipore, Cat# IPVH00010). Membranes were blocked with 5% BSA (Solarbio, Cat# A8010) at room temperature for 1 hour (alternatively, 5% skim milk in TBST; BD Difco, Cat# 232100), then incubated overnight at 4 °C with primary antibodies diluted according to manufacturers’ instructions (**Supplementary Table 1**). HRP-conjugated secondary antibodies were applied for 1 hour at room temperature, and signals were developed using ECL substrate (Thermo Fisher, Cat# 32106). GAPDH was used as the internal control, and signal intensities were quantified using ImageJ (NIH, USA) after background subtraction and normalization.

### IF staining

Both paraffin-embedded and OCT-embedded frozen sections (8 μm) were processed using a standardized protocol. Sections were deparaffinized in xylene, rehydrated through a graded ethanol series, and subjected to heat-induced epitope retrieval using EDTA buffer (pH 9.0; Servicebio, Cat# G1206) at 95-98 °C for 15 minutes. Permeabilization was performed with 0.3% Triton X-100 for 10 minutes, then blocking with 5% BSA for 30 minutes at room temperature. Primary antibodies were incubated overnight at 4 °C, then 1-hour incubation with species-specific fluorescent secondary antibodies (Invitrogen, A-11001/A-11008) at room temperature. Nuclei were counterstained with DAPI (1 μg/mL; Invitrogen, D1306) for 5 minutes, and slides were mounted with antifade mounting medium (Vector, H-1200). Images were acquired using a Leica TCS SP8 confocal microscope under fixed excitation, gain, and exposure settings. At least five non-overlapping fields per section were captured, with z-stacks processed under uniform parameters in FIJI. Primary antibody details are listed in **Supplementary Table 2**.

### Immunofluorescence co-localization staining and quantitative analysis

Paraffin-embedded TC tissue sections were deparaffinized, subjected to antigen retrieval, and blocked before overnight incubation at 4 °C with mixed primary antibodies against TREM2 (rabbit anti-TREM2; Abcam, ab209814, 1:200) and CD163 (mouse anti-CD163; Abcam, ab182422, 1:200). After PBS washing, sections were incubated with Alexa Fluor 594-conjugated goat anti-rabbit IgG (Invitrogen, A-11012, 1:500) and Alexa Fluor 488-conjugated goat anti-mouse IgG (Invitrogen, A-11001, 1:500) for 1 hour at room temperature in the dark. After DAPI mounting, images were acquired using a Leica TCS SP8 confocal microscope, and five non-overlapping high-power fields (x400) were randomly selected for each sample. Co-localization was quantified with the Coloc 2 plugin in ImageJ/Fiji using Otsu threshold segmentation for red and green channels and Costes automatic threshold calibration. Pearson correlation coefficients (PCCs) were calculated; values greater than 0.5 were considered to indicate strong co-localization, and results are reported as the mean ± SD of five fields.

### Double immunofluorescence staining and quantitative analysis of cell functional markers

To evaluate the cytotoxic function of CD8^+^ T cells, paraffin sections were subjected to double immunofluorescence staining for CD8 and Granzyme B. Sections were deparaffinized, antigen-retrieved in EDTA buffer (pH 9.0, 95-98 °C, 15 minutes), permeabilized with 0.3% Triton X-100 for 10 minutes, and blocked before overnight incubation at 4 °C with rabbit anti-CD8 (CST, 85336, 1:200) and mouse anti-Granzyme B primary antibodies. After PBS washing, the corresponding Alexa Fluor 488-conjugated goat anti-rabbit IgG and Alexa Fluor 594-conjugated goat anti-mouse IgG secondary antibodies were applied for 1 hour at room temperature in the dark. Nuclei were counterstained with DAPI, and sections were mounted with antifade medium. Images were acquired using a Leica TCS SP8 confocal microscope under identical acquisition settings, with at least five non-overlapping high-power fields (x400) randomly selected for each sample. Quantification was performed in ImageJ/Fiji by creating a binary mask based on CD8-positive signals to define CD8^+^ T cell regions, followed by measurement of Granzyme B fluorescence intensity and positive area within this mask. The percentage of Granzyme B-positive area within the total CD8^+^ mask area was used as an index of CD8^+^ T cell cytotoxic function.

### RT-qPCR

Total RNA was extracted using TRIzol reagent (Invitrogen, 15596026), and genomic DNA was removed using RNase-free DNase I (Takara, 2270A). RNA purity was assessed by NanoDrop, with acceptable A260/280 ratios between 1.8 and 2.0. cDNA synthesis was performed with the PrimeScript RT reagent kit (Takara, RR037A), and quantitative PCR (qPCR) was carried out using SYBR Green (Takara, RR420A) on a QuantStudio 6 system (Thermo Fisher). GAPDH served as the internal reference gene. Relative expression levels were calculated with the 2-ΔΔCt method. Melt curves verified specificity, and amplification efficiency ranged from 90% to 110%. Primer sequences are provided in **Supplementary Table 3**.

### Chromatin immunoprecipitation-quantitative polymerase chain reaction

Sorted macrophages were crosslinked with 1% formaldehyde (Sigma-Aldrich, Cat# F8775) for 10 minutes, and the reaction was quenched with 0.125 M glycine (Sigma-Aldrich, Cat# G8898) for 5 minutes. Chromatin was sheared to 200-500 bp fragments using a Bioruptor Pico sonicator (Diagenode) with 30 s on/30 s off cycles for 10 minutes. Immunoprecipitation was performed with the Magna ChIP A/G kit (Millipore, 17-10085) with either 5 μg anti-AHR antibody (CST, 83200) or an IgG control, then overnight incubation at 4 °C. Samples were sequentially washed with low-salt, high-salt, and TE buffers, then reverse crosslinked overnight at 65 °C. After RNase and Proteinase K treatment, DNA was purified using a QIAquick PCR Purification Kit (Qiagen, 28106). qPCR was carried out targeting XRE regions within the Ido1 promoter (-500 to -200 bp relative to the TSS), using TB Green (Takara). Results were expressed as % input and fold enrichment relative to IgG controls.

### *In vitro* functional assays of macrophage immunoregulation

Bone marrow-derived macrophages (BMDMs) were generated by culturing femur and tibia bone marrow cells in RPMI-1640 medium (Gibco, 11875093) supplemented with 10% fetal bovine serum (FBS; Gibco, 26140079), 1% penicillin-streptomycin (Gibco, 15140122), and granulocyte-macrophage colony-stimulating factor (GM-CSF; 20 ng/mL, PeproTech, 315-03) for 7 days, with medium changes every other day. On day 7, cells were polarized with IL-4 (10 ng/mL) and TGF-β (5 ng/mL; PeproTech) for 48 hours, then flow cytometric sorting into TREM2^+^ and TREM2- populations (Abcam, ab209814). These macrophages were then co-cultured with CD8^+^ T cells (Miltenyi, 130-104-075; purity >95%) at a 1:4 macrophage-to-T cell ratio in 24-well plates for 24–48 hours (**Supplementary Figure 3**).

*In vitro* experimental grouping: Baseline groups included CD8^+^ T cells cultured alone (Control), co-cultured with TREM2- macrophages, or with TREM2^+^ macrophages.

Pharmacological intervention groups were as follows: Vehicle: 0.1% DMSO; AHR inhibition: CH223191, 10 μM for 24–48 hours (Selleckchem, Cat# S7711); IDO1 inhibition: 1-MT, 500 μM for 24–48 hours (Sigma-Aldrich, Cat# M8377); Combination: CH223191 10 μM + 1-MT 500 μM.

AHR activation/rescue assays: Kyn stimulation (T cell side): 20 μM for 24 hours (Sigma-Aldrich, Cat# K8625).

Treg function enhancement: Kyn 5 μM applied to Tregs for 24 hours.

### LC-MS/MS quantification of Kyn in tumor tissues

Tumor tissues (20-30 mg) were placed in pre-chilled homogenization tubes with four volumes of 80% methanol containing an internal standard ([13C6]-Kyn, 50 ng/mL; Toronto Research Chemicals, Fisher Chemical, Cat# A456-4). Homogenization was carried out on ice using the Precellys Evolution system (6,500 rpm, 2 × 20 s). Protein precipitation was carried out at -20 °C for 20 minutes, then centrifugation at 12,000 × g for 10 minutes at 4 °C. The supernatant was filtered through a 0.22 μm membrane (Millipore, SLGV033RS). Quantification was performed with UPLC (Waters ACQUITY H-Class) coupled with a triple quadrupole mass spectrometer (TQ-XS). Chromatographic separation was achieved on an HSS T3 column (2.1 × 100 mm, 1.8 μm) with mobile phase A (0.1% formic acid in water) and B (0.1% formic acid in acetonitrile). The gradient was as follows: 0-2 min, 2% B; 2-6 min, linear increase to 40% B; 6-7 min to 95% B; 7-9 min hold at 95% B; flow rate: 0.30 mL/min. Kyn was detected via MRM transition m/z 209→94 (collision energy: 20 eV). External standards (1-1000 ng/mL) served to construct calibration curves (R2 ≥ 0.995), with a lower limit of quantification of 5 ng/mL. Results were reported as pmol/mg tissue. One quality control (QC) sample was included every 10 injections, with intra- and inter-batch RSDs below 15%. For cell supernatants, the same sample preparation was applied, and quantification was performed with isotope-labeled internal standards and the external calibration curve.

### IDO1 enzymatic activity assay

IDO1 activity was assessed using a colorimetric assay kit (BioVision, Cat# K995) according to the manufacturer’s protocol. Briefly, tumor lysates (total protein concentration: 1 mg/mL, prepared in RIPA buffer [Beyotime, Cat# P0013B] with protease inhibitors) were incubated in a 100 μL reaction system containing L-tryptophan substrate at 37 °C for 60 minutes. After incubation, absorbance was measured at 490 nm (reference wavelength: 650 nm) using a microplate reader (SpectraMax iD3, Molecular Devices). Kyn production was calculated on the basis of a standard curve (0-100 nmol/well, R2 ≥ 0.99). IDO1 activity was expressed as the rate of Kyn production, normalized to protein content, and reported as µmol/min/mg protein.

### Statistical analysis

Statistical analyses were performed with GraphPad Prism and R. Data were presented as mean ± standard deviation (SD), with n indicating the number of independent biological replicates. Normality was assessed before testing. For two-group comparisons, two-tailed t tests were used (Mann-Whitney U test if non-normal). For multiple groups, one-way analysis of variance (ANOVA) with Tukey’s *post hoc* test was applied (Kruskal-Wallis with Dunn’s test for non-parametric data). A two-way ANOVA was applied for experiments with two independent variables. Correlation analyses employed Pearson or Spearman methods. For multiple comparisons, p-values were adjusted using the Benjamini-Hochberg method. Statistical significance was defined as *p* < 0.05 (FDR < 0.05 for omics analyses).

## Results

### Aberrant enrichment of TREM2^+^ macrophages in the TC microenvironment and their positive correlation with tumor progression

**Figure 1:**
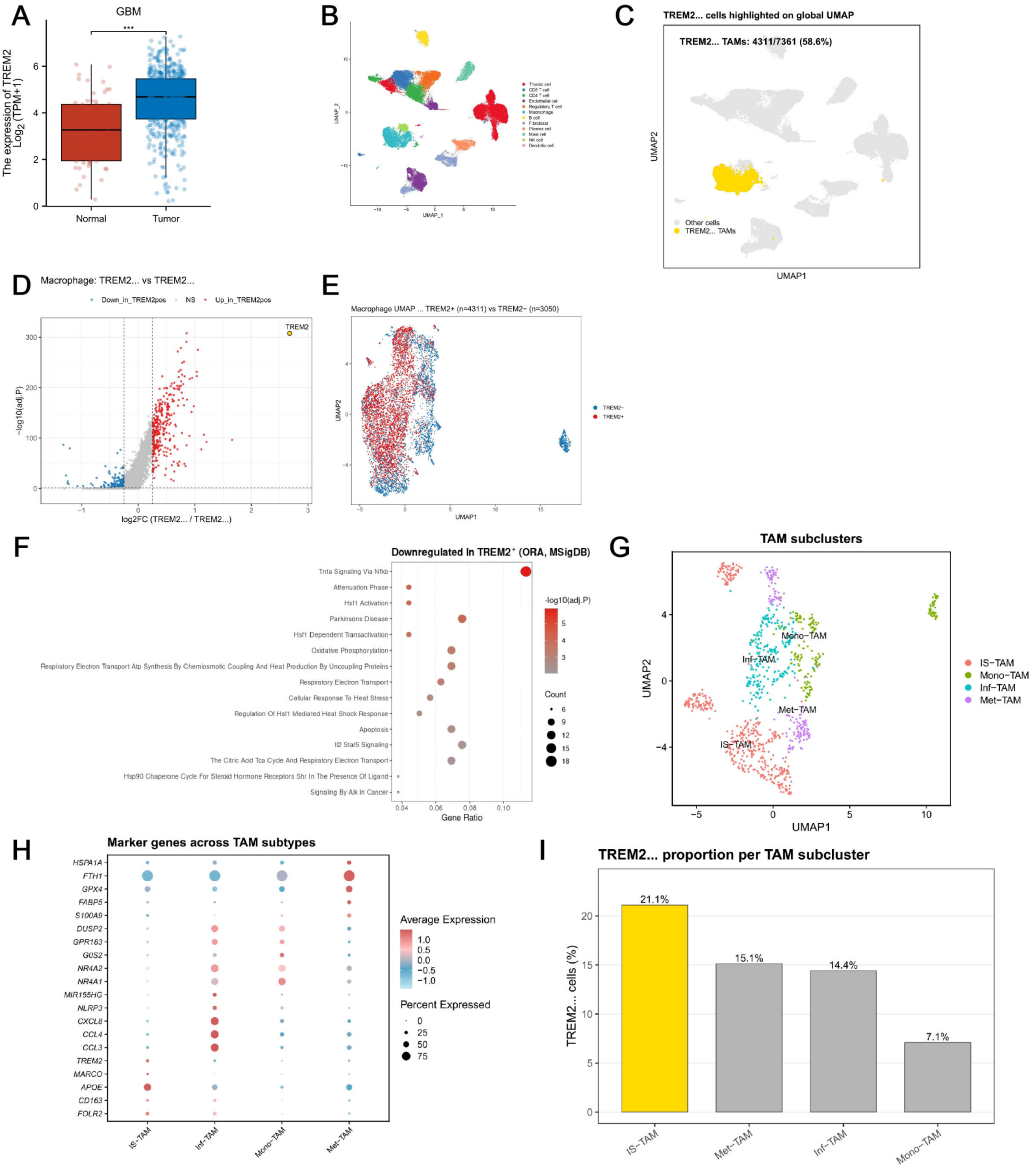
Expression profile and clinical relevance of TREM2^+^ macrophages in TC. **(A)** Integrated TCGA-THCA analysis shows that TREM2 expression is significantly elevated in TC tissues relative to normal tissues (log2FC = 0.49, *p* < 0.0001), Normal (n = 58); **(B)** UMAP plot of integrated single-cell data with cell lineage annotation; tumor group: 6 samples, normal group: 3 samples; **(C)** TREM2 is highly preferentially enriched in TAMs within the TC microenvironment. UMAP visualization indicates a distinct TAM subset (yellow), of which TREM2-positive TAMs (blue) constitute 58.6% (4311 out of 7361 cells) of the TAM population; **(D)** Volcano plot comparing TREM2^+^ and TREM2- subsets within TAMs, with TREM2 highlighted; **(E)** UMAP plot of TAM subsets colored by TREM2^+^ and TREM2- cells, showing an interwoven distribution within the same manifold; **(F)** Overrepresentation analysis of pathways altered in TREM2^+^ TAMs; **(G)** UMAP visualization of TAM subclusters after reclustering; **(H)** marker-gene expression patterns for the four TAM subclusters; **(I)** quantification of the distribution of TREM2^+^ cells across TAM subclusters.

In the TCGA-THCA cohort, TREM2 expression was significantly elevated in TC tissues (**Figure 1A**). This observation was independently confirmed using the publicly available GSE232237 dataset, which includes three normal and six tumor samples. In this scRNA-seq dataset, cells were first annotated using the reference framework and canonical markers described by Lee et al. (25) (**Figure 1B**). Global UMAP visualization showed that TREM2^+^ cells were almost exclusively localized within the TAM compartment, accounting for 58.6% (4311/7361) of TAMs (**Figure 1C**), suggesting TREM2 expression is lineage-specific.

Differential analysis between TREM2+ and TREM2- TAMs identified extensive transcriptional remodeling, with TREM2 itself markedly increased in the TREM2+ subset (**Figure 1D**). Spatial mapping indicated that TREM2+ and TREM2- cells were interspersed along a shared macrophage trajectory (**Figure 1E**), which indicates that TREM2 expression reflects a continuum of functional states rather than defining a discrete macrophage subpopulation. Pathway enrichment analysis showed significant downregulation of mitochondrial oxidative phosphorylation, respiratory chain components, and the TCA cycle, as well as inflammation- and stress-related pathways, including TNF-α-NF-κB, IL-2-STAT5, and heat shock responses in TREM2+ cells (**Figure 1F**), pointing toward an immunosuppressive phenotype accompanied by metabolic reprogramming.

To further investigate TAM heterogeneity and its relationship with TREM2 expression, TAMs were reclustered and four major subgroups were identified: immunosuppressive TAMs (IS-TAMs), monocyte-like TAMs (Mono-TAMs), inflammatory TAMs (Inf-TAMs), and metabolic TAMs (Met-TAMs) (**Figure 1G**). These TAM subgroups displayed distinct marker-gene expression patterns (**Figure 1H**). Quantitative assessment of the distribution of TREM2^+^ cells across TAM subgroups showed that TREM2^+^ cells were most abundant in the IS-TAM subgroup (21.1%), followed by Met-TAMs (15.1%) and Inf-TAMs (14.4%), whereas the lowest proportion was observed in Mono-TAMs (7.1%) (**Figure 1I**). These findings further support the preferential enrichment of TREM2 expression in specific functional states of TAMs.

### Spatial distribution of TREM2^+^ TAMs at the infiltrative margin and their immunological interaction with CD8^+^ T cells

**Figure 2:**
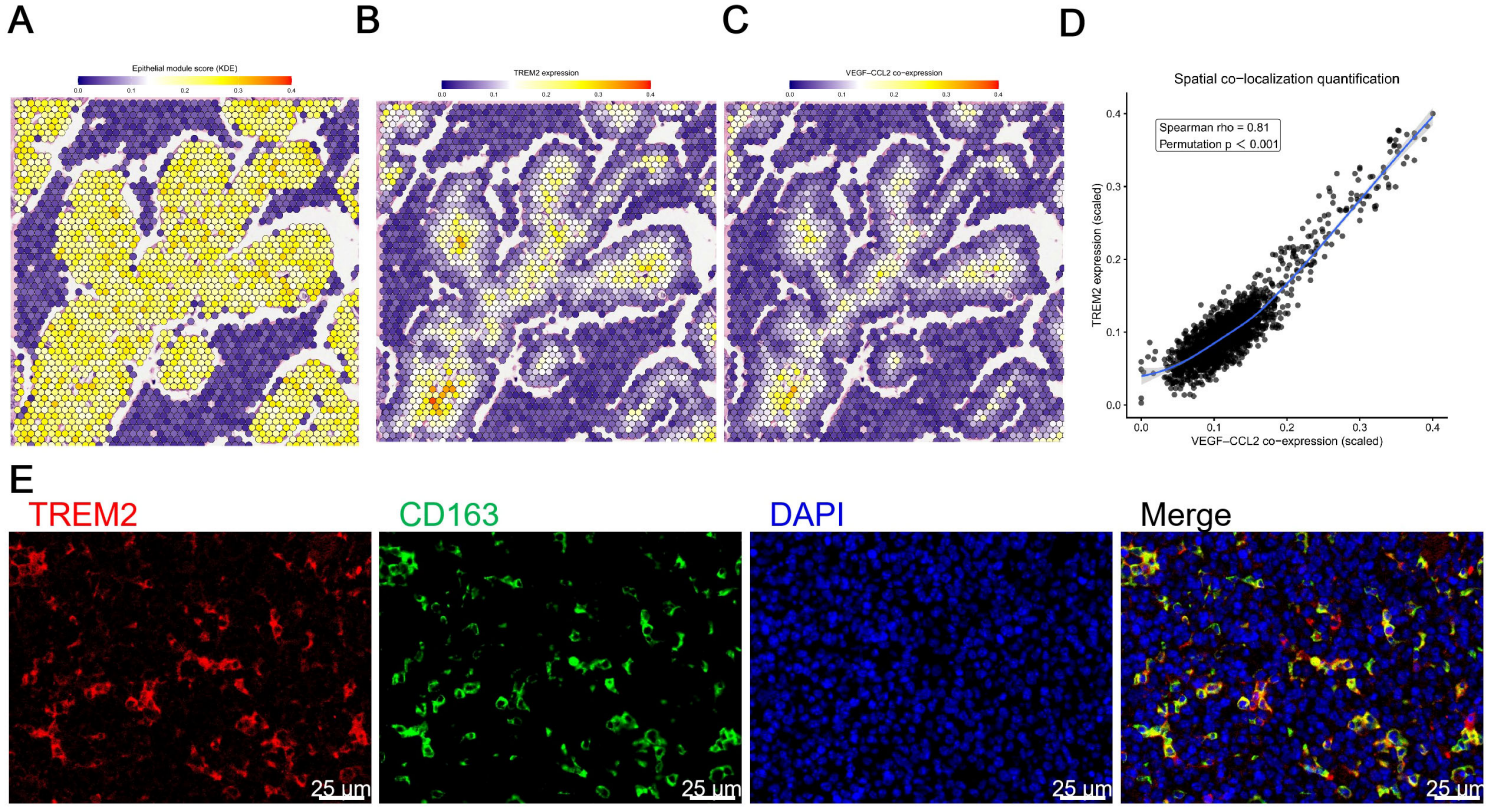
Expression profile and clinical association of TREM2^+^ macrophages in TC. **(A)** Kernel density estimation (KDE) on the basis of epithelial-associated genes TG, EPCAM, KRT8, and KRT18 delineates the tumor epithelial regions; **(B)** spatial enrichment pattern of TREM2 in tumor tissues, with hotspots primarily localized to regions related to immunosuppression; **(C)** co-expression hotspots of VEGF and CCL2 partially overlap with both tumor epithelial zones and TREM2-enriched areas; **(D)** quantitative correlation analysis between VEGF/CCL2 co-expression hotspots and TREM2-enriched regions; **(E)** double immunofluorescence staining showing co-localization of TREM2 (red) and CD163 (green) in tumor stroma, with yellow signal in the merged image indicating double-positive cells. Nuclei were counterstained with DAPI (blue). Bar = 25 μm.

To further elucidate the spatial distribution of TREM2, IF staining was performed on TC tissue sections. To precisely delineate tumor regions, epithelial-associated genes (TG, EPCAM, KRT8, and KRT18) were selected as molecular markers. These genes are widely used in histological and molecular studies of thyroid and epithelial-derived tumors and effectively distinguish tumor epithelium from surrounding stroma or immune compartments (**Figure 2A**). Based on this framework, spatial enrichment analysis revealed that TREM2 signals were predominantly concentrated in discrete intratumoral regions, exhibiting patchy or island-like distributions. These areas were closely associated with immunosuppressive microenvironments, suggesting a potential role of TREM2 in local immune modulation (**Figure 2B**). Regions co-expressing VEGF and CCL2 showed strong spatial overlap with TREM2-enriched zones, reinforcing the potential association between TREM2 and immunosuppressive pathways (**Figure 2C**). Quantitative spatial transcriptomic analysis further showed that VEGF-CCL2 co-expression intensity was positively correlated with TREM2 expression across matched spots (Spearman r = 0.81, *p* < 0.001) (**Figure 2D**). Double immunofluorescence staining demonstrated marked co-localization of TREM2 with the M2 macrophage marker CD163 within the tumor stroma (**Figure 2E**), confirming that TREM2^+^ macrophages exhibit a classical immunosuppressive phenotype.

Together, these results suggest that TREM2^+^ TAMs are preferentially enriched in spatially defined immunosuppressive niches in TC and may contribute to tumor progression and immune evasion through their association with VEGF/CCL2-rich regions and CD163+ M2-like macrophage programs.

### Identification and functional validation of a distinct TREM2^+^ macrophage subpopulation

**Figure 3:**
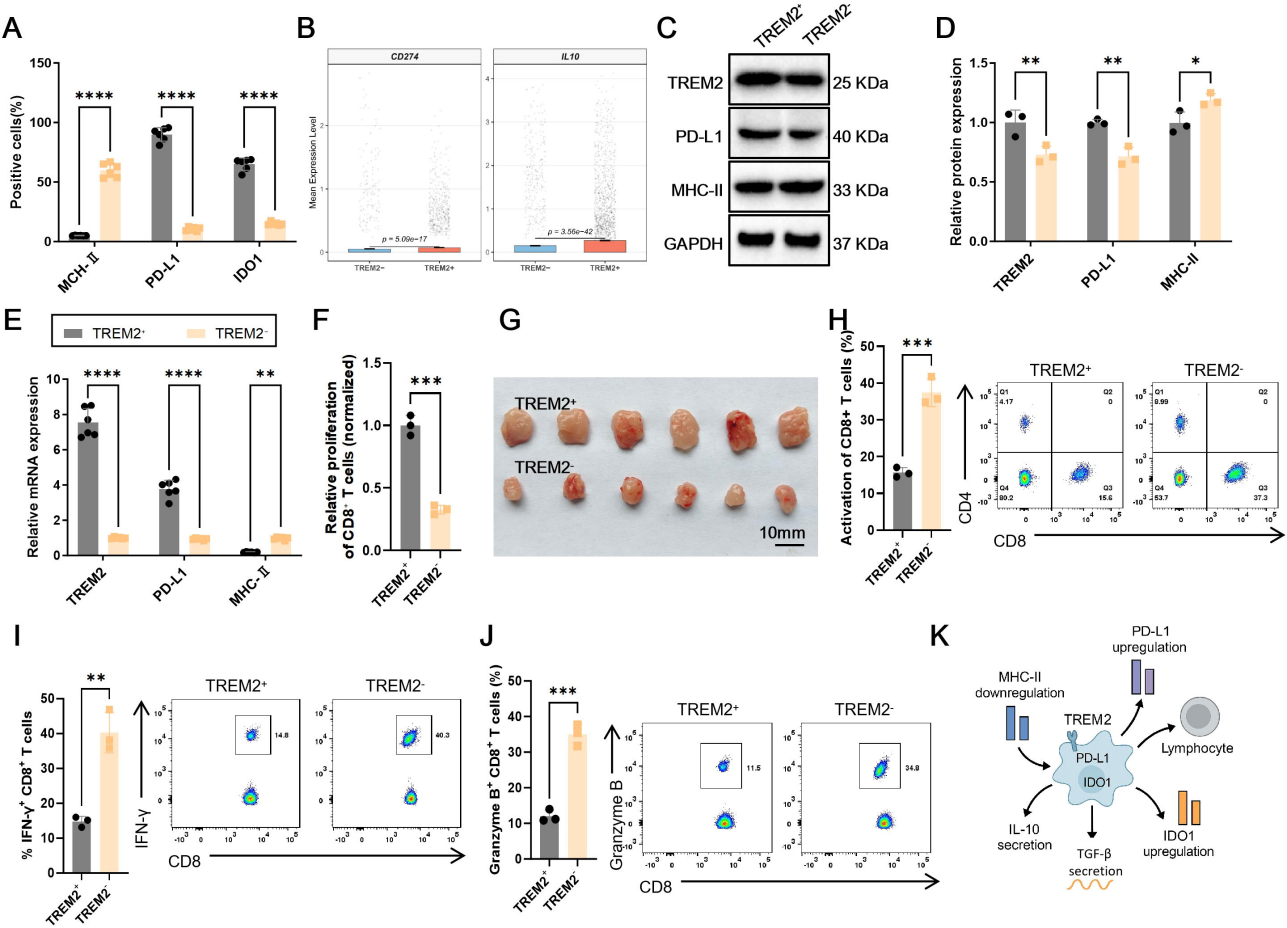
Phenotypic characterization and functional validation of TREM2^+^ macrophages. **(A)** Mass cytometry (CyTOF) analysis of MHC-II, PD-L1, and IDO1 positivity in TREM2^+^ and TREM2- macrophages isolated from murine TC tissues; **(B)** transcriptomic profiling of immune-related genes CD274 (PD-L1) and IL-10 in TREM2^+^ versus TREM2- macrophages; **(C)** Western blot analysis of TREM2, PD-L1, and MHC-II protein levels in TREM2^+^ and TREM2- macrophages; **(D)** quantification of relative protein expression via grayscale analysis; **(E)** RT-qPCR quantification of TREM2, PD-L1, and MHC-II mRNA expression levels; **(F)** assessment of CD8^+^ T cell proliferation; **(G)** gross examination of tumor size across mouse groups, scale bar = 10 mm; **(H)** flow cytometric analysis of CD8^+^ T cell activation; **(I)** flow cytometric measurement of IFN-γ production in CD8^+^ T cells; **(J)** flow cytometric detection of Granzyme B expression in CD8^+^ T cells; **(K)** schematic illustration of the immunoregulatory mechanism mediated by TREM2^+^ macrophages in TC. All cell-based experiments were conducted in triplicate. Each animal group included six mice. ns, *p* > 0.05; *p < 0.05; **p < 0.01; ***p < 0.001; ****p < 0.0001.

To further characterize the phenotypic and functional properties of TREM2^+^ macrophages in TC, we investigated whether this subset differs from classical M1/M2 macrophages and possesses unique immunoregulatory features. CyTOF showed that, relative to the TREM2- macrophage group, TREM2^+^ macrophages exhibited significantly reduced MHC-II expression, suggesting a compromised antigen-presenting capacity. In contrast, PD-L1 and IDO1 levels were markedly elevated, which indicates that TREM2^+^ macrophages adopt a prototypical immunosuppressive phenotype, potentially contributing to immune evasion within the tumor microenvironment (**Figure 3A**).

Phenotypic analysis further verified that immunosuppressive molecules were substantially increased in the TREM2^+^ macrophage population. Specifically, TREM2^+^ TAMs showed significantly increased expression of PD-L1 (CD274) and IL-10 relative to their TREM2- counterparts (**Figure 3B**). These findings imply that this subpopulation may exert enhanced immunosuppressive effects by elevating immune checkpoint molecules and cytokines, thereby promoting immune evasion in the tumor microenvironment. The molecular profile of this subset was then confirmed at both the protein and mRNA levels. Western blot and RT-qPCR analyses consistently indicated that, relative to the TREM2- group, TREM2^+^ macrophages displayed significantly higher expression of TREM2 and PD-L1, alongside reduced expression of MHC-II (**Figures 3C–E**). Notably, a detectable TREM2 protein band remained in the TREM2- group, consistent with the continuum of TREM2 expression revealed by the single-cell data (**Figure 1E**); densitometric quantification confirmed that TREM2 protein levels were significantly higher in the TREM2^+^ group.

In the *in vitro* co-culture assay, TREM2^+^ macrophages showed a significantly stronger capacity to suppress CD8^+^ T cell proliferation relative to TREM2- macrophages, supporting their immunosuppressive function (**Figure 3F**). In a murine TC model, we further compared the effects of TREM2^+^ and TREM2- macrophages on tumor growth and CD8^+^ T cell activity. Macroscopically, mice in the TREM2- group developed visibly smaller tumors than those in the TREM2^+^ group (**Figure 3G**), which suggests that blockade of TREM2 signaling may effectively inhibit tumor progression. Additionally, CD8^+^ T cells isolated from the TREM2- macrophage group exhibited markedly higher activation levels than those from the TREM2^+^ group (**Figure 3H**). Moreover, the proportion of IFN-γ+ CD8^+^ T cells was significantly elevated in the TREM2- macrophage group relative to the TREM2^+^ group (**Figure 3I**), and a similar increase was observed in the proportion of Granzyme B^+^ CD8^+^ T cells (**Figure 3J**). The schematic model in **Figure 3K** summarizes these findings, showing that TREM2^+^ macrophages promote immune evasion by impairing antigen presentation, upregulating PD-L1/IDO1-associated immunosuppressive programs, and suppressing CD8^+^ T cell effector activity. These findings indicate that TREM2- macrophages enhance the activation and cytotoxic function of CD8^+^ T cells, thereby exerting an anti-tumor effect within the tumor microenvironment.

Collectively, TREM2^+^ macrophages facilitate tumor immune evasion and disease progression by impairing antigen presentation, upregulating immune checkpoint molecules, and secreting immunosuppressive cytokines.

### Multi-omics analysis reveals functional activation of the AHR axis in TREM2^+^ macrophages

**Figure 4:**
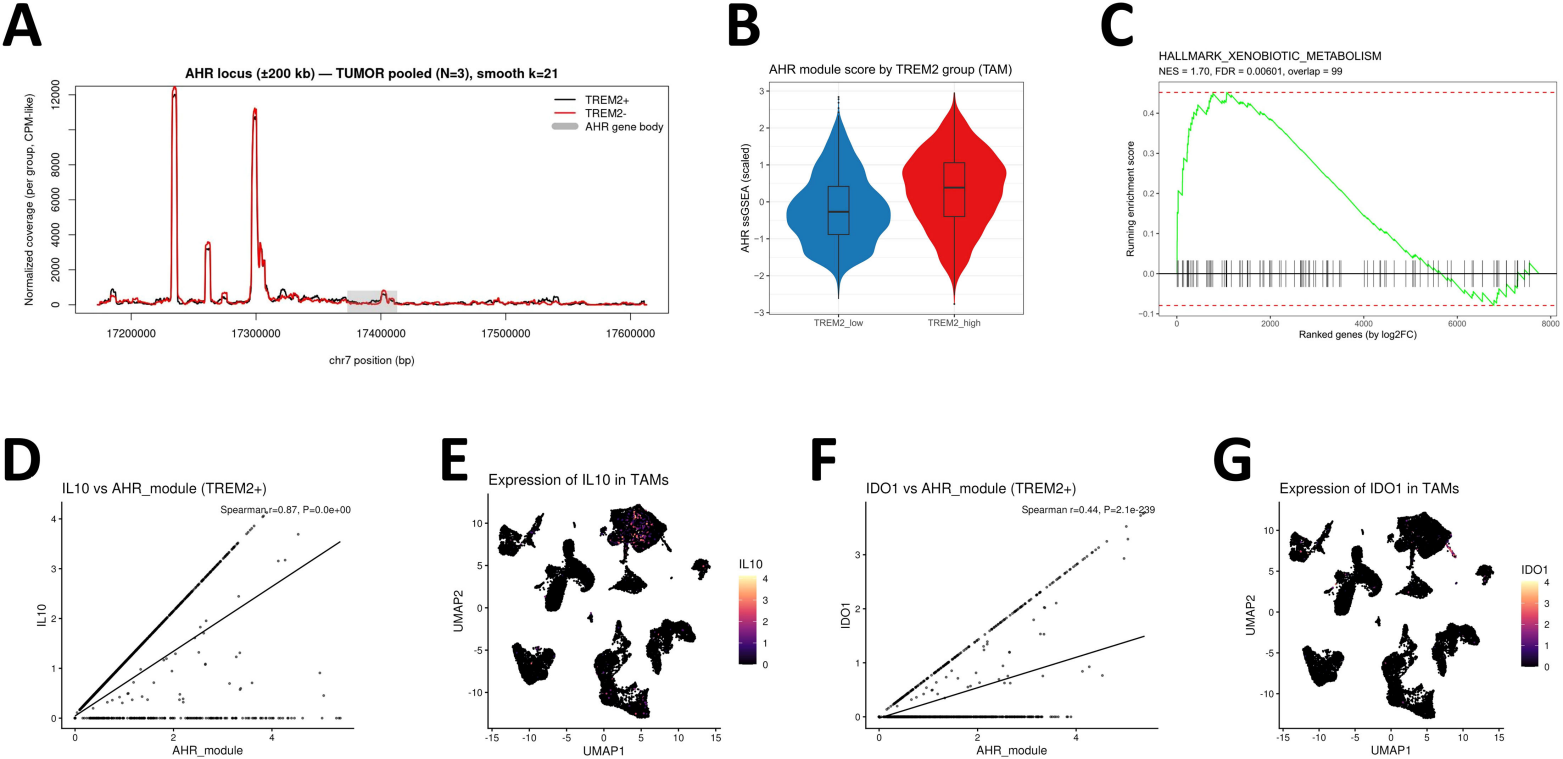
Chromatin accessibility and activation of AHR signaling in TREM2^+^ macrophages. **(A)** Chromatin accessibility at the mouse Ahr locus in TAMs isolated from murine thyroid cancer tissues, as assessed by scATAC-seq; **(B)** AHR module score distribution in TREM2+ and TREM2- TAMs calculated using the ssGSEA approach; **(C)** GSEA results showing enrichment of AHR target gene sets in TREM2+ TAMs; **(D)** Spearman correlation analysis between AHR module scores and IL10 expression levels; **(E)** FeaturePlot of scRNA-seq data visualizing IL10 expression in TREM2+ TAMs; **(F)** Spearman correlation between AHR module scores and IDO1 expression; **(G)** FeaturePlot illustrating the spatial distribution of IDO1 expression in TREM2+ TAMs on the basis of scRNA-seq data.

To elucidate the upstream regulatory mechanisms of TREM2+ macrophages within the TC microenvironment, we first compared the chromatin accessibility profiles of TREM2+ and TREM2- macrophages using scATAC-seq. Visualization of chromatin accessibility across a ±200 kb region surrounding the AHR locus identified largely similar global profiles between the two populations. However, a slightly elevated accessibility peak was observed in TREM2+ macrophages at a localized window (chr7:1.739-1.741 Mb), suggesting potential site-specific chromatin opening near AHR (**Figure 4A**), indicative of enhanced accessibility in the vicinity of AHR in TREM2+ cells.

Functional enrichment analysis at the single-cell level identified markedly elevated AHR pathway activity in TREM2high TAMs. Based on ssGSEA scoring, the AHR module scores were consistently higher in the TREM2high group than in the TREM2low group, indicating a strong association between TREM2 expression levels and AHR signaling activity (**Figure 4B**). This finding was further supported by GSEA, which showed significant enrichment of canonical AHR target genes (IDO1, CYP1A1, IL10) in TREM2high TAMs (NES = 1.70, FDR = 0.00601), reflecting robust activation of AHR downstream pathways in this population (**Figure 4C**). Together, these results suggest that TREM2high TAMs may leverage enhanced AHR signaling to drive the expression of immunosuppressive genes, thereby contributing to their immunoregulatory capacity and potential to promote immune evasion.

Further Spearman correlation analysis identified a strong positive correlation between the AHR module score and the expression of downstream targets IL10 (r = 0.87, p < 0.001) and IDO1 (r = 0.44, p < 0.001), supporting a direct transcriptional regulatory role for AHR (**Figure 4D** and 4F). Notably, single-cell expression mapping showed considerable heterogeneity in the expression of immunosuppressive markers IL10 and IDO1 among TAMs, with some cells displaying significantly higher levels than the population average (**Figures 4E, G**), suggesting the presence of immunosuppressive subpopulations within the TAM compartment.

In summary, TREM2^+^ macrophages exhibited locally increased chromatin accessibility near the AHR locus, accompanied by enhanced activity of AHR-associated signaling pathways and upregulation of immunoregulatory genes. Collectively, these findings implicate AHR as a potentially druggable regulatory node in TREM2^+^ macrophages.

### Functional validation of metabolic reprogramming and immunosuppression mediated by the Kyn pathway

To investigate the role of TREM2^+^ macrophages in immunometabolic regulation within the TC microenvironment, we quantified Kyn accumulation and IDO1 enzymatic activity in TREM2-defined tumor regions.

**Figure 5:**
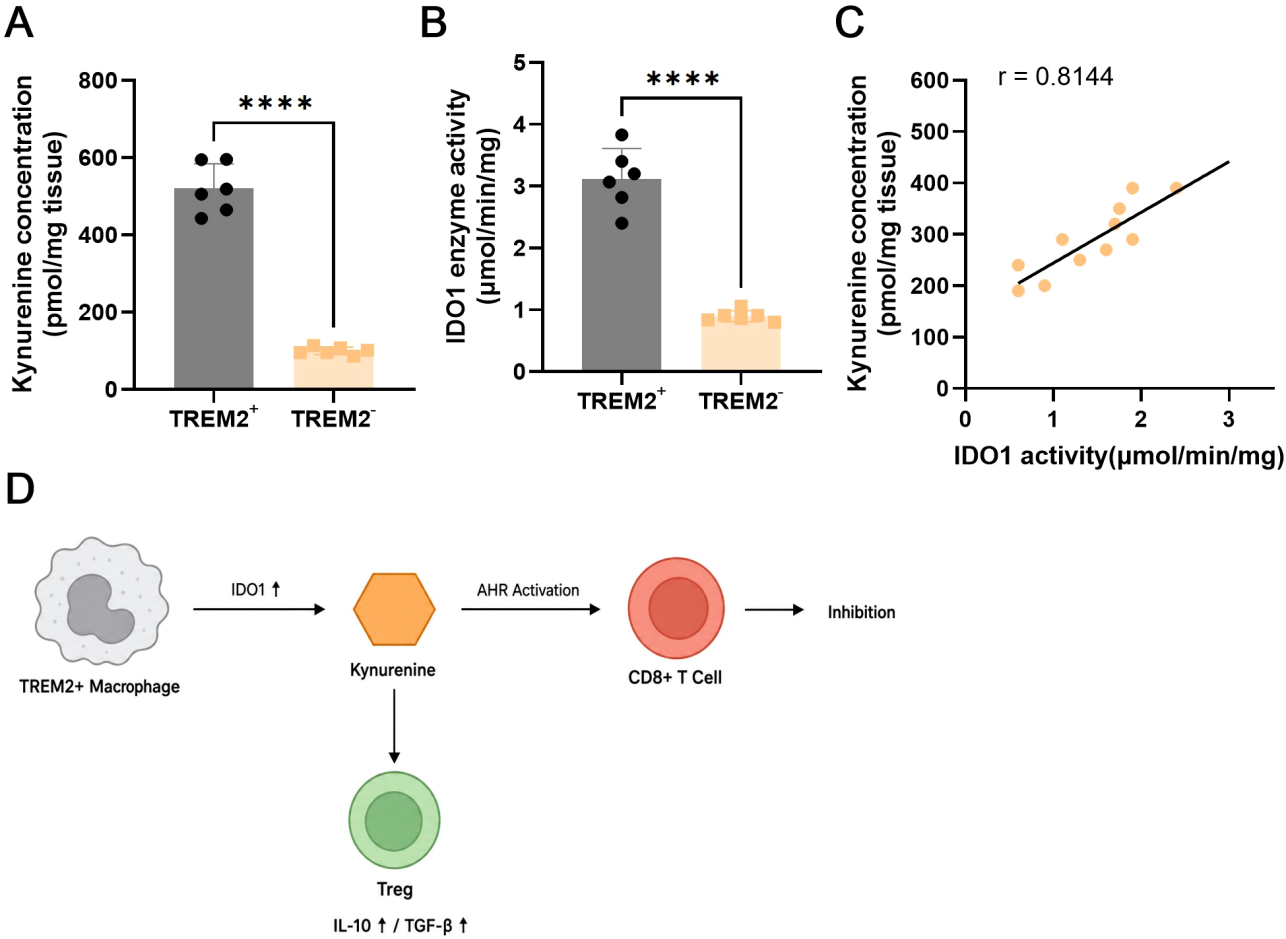
Activation of the Kyn metabolic pathway in TREM2^+^ macrophage-enriched regions. **(A)** Quantification of Kyn concentration in tissue samples by LC-MS/MS; **(B)** enzymatic assays comparing IDO1 activity in TREM2^+^ versus TREM2- regions; **(C)** Spearman correlation analysis between IDO1 enzymatic activity and Kyn concentration; **(D)** schematic model of the IDO1-Kyn metabolic axis, illustrating how TREM2^+^ macrophages upregulate IDO1 activity to enhance Kyn synthesis, thereby contributing to CD8^+^ T cell suppression and immune exhaustion. Each group consisted of six animals; experiments were repeated three times. ****p < 0.0001.

We initially performed TREM2 immunohistochemical staining on serial tumor sections, then digital scanning. Using an image analysis system, we delineated TREM2high and TREM2low regions within the same tumor on the basis of staining intensity thresholds. Corresponding areas on adjacent unstained sections were then microdissected using MMD/LCM for metabolic and enzymatic analyses. Quantification of tissue Kyn levels via LC-MS/MS identified significantly higher concentrations in TREM2high regions than in TREM2low regions (**Figure 5A**), suggesting potential accumulation of this metabolite within immunosuppressive niches.

To determine the enzymatic source driving elevated Kyn levels, we assessed IDO1 activity and found it to be markedly increased in TREM2^+^ regions relative to TREM2- regions (**Figure 5B**). Correlation analysis further showed a positive association between IDO1 activity and Kyn concentration, supporting IDO1 as a key enzymatic driver of Kyn metabolism (**Figure 5C**). The schematic model in **Figure 5D** illustrates that TREM2^+^ macrophages upregulate IDO1 activity to enhance Kyn synthesis, thereby contributing to CD8^+^ T cell suppression and immune exhaustion.

Together, these findings suggest that activation of the IDO1-Kyn axis is tightly linked to the enrichment of TREM2^+^ macrophages and may represent a central mechanism by which they induce immune tolerance and facilitate tumor immune evasion. This provides experimental evidence supporting the potential of targeting this metabolic pathway for immunotherapeutic intervention.

### Dual inhibition of AHR and IDO1 restores CD8^+^ T cell effector function

**Figure 6:**
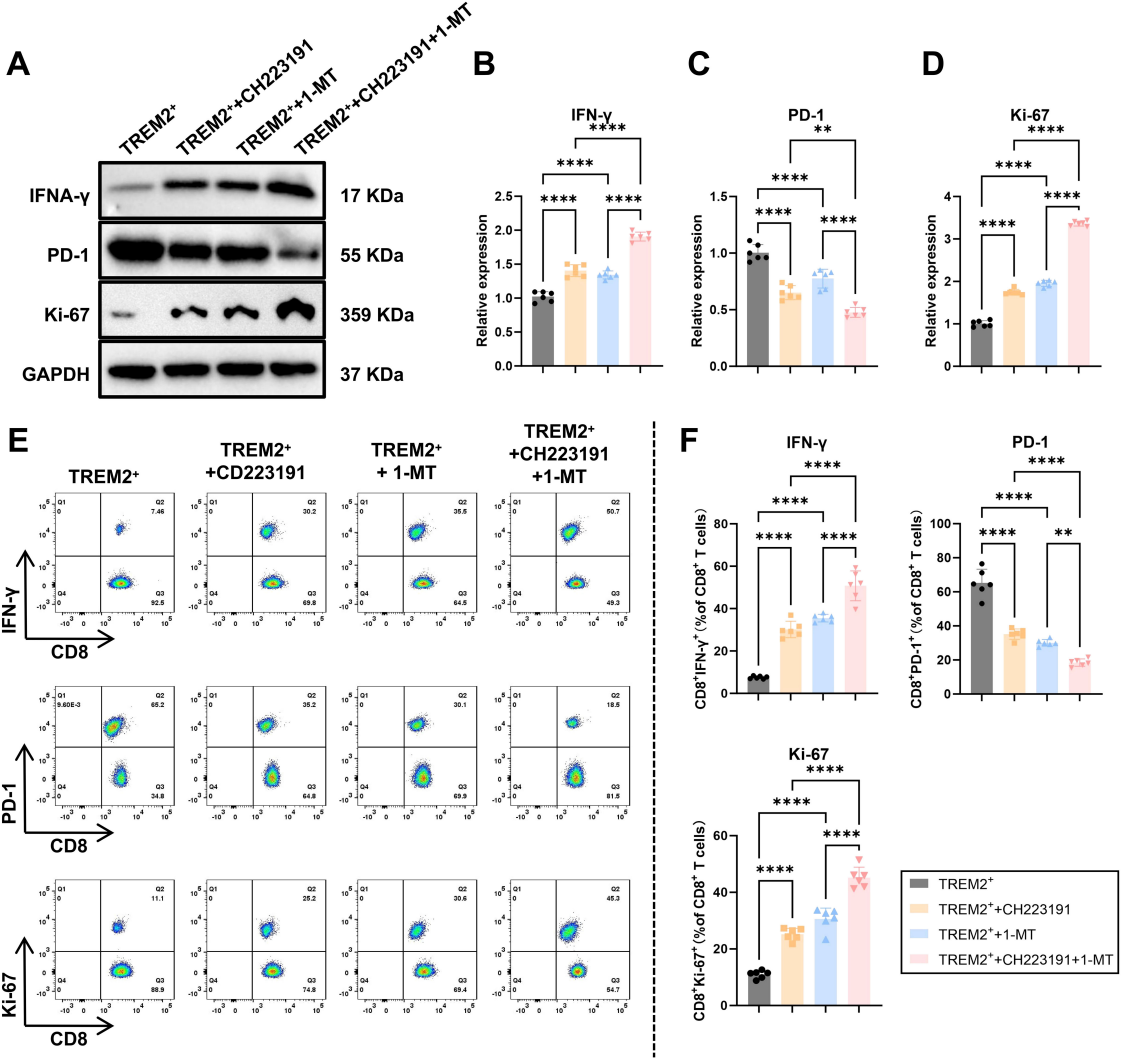
Combined blockade of AHR and IDO1 restores CD8^+^ T cell effector function. **(A)** Western blot analysis of IFN-γ, PD-1, and Ki-67 protein expression in TREM2^+^ tumor tissues; **(B–D)** Quantitative analysis of protein expression levels in tumor tissues using densitometric evaluation of Western blot bands for IFN-γ **(B)**, PD-1 **(C)**, and Ki-67 **(D)**; **(E)** Multicolor flow cytometry (gated on CD8^+^) assessing the expression patterns of IFN-γ, PD-1, and Ki-67 in tumor-infiltrating lymphocytes (TILs); **(F)** Statistical analysis of the proportions of CD8^+^IFN-γ+, CD8^+^PD-1^+^, and CD8^+^Ki-67^+^ cells within TILs, reflecting the activation and proliferative status of CD8^+^ T cells. Each group included six mice. **p < 0.01; ****p < 0.0001.

To evaluate whether dual inhibition of AHR and IDO1 could effectively restore CD8^+^ T cell effector function, we conducted *in vivo* pharmacological intervention in a TREM2^+^ tumor model. Changes in key effector molecules were assessed via Western blot and flow cytometry to compare the effects of monotherapy and combination therapy. First, Western blot analysis was carried out to quantify the protein levels of IFN-γ, PD-1, and Ki-67 in tumor tissues (**Figure 6A**), then densitometric quantification (**Figures 6B–D**). Compared to the TREM2^+^ group, the TREM2^+^ + CH223191 group exhibited significantly increased levels of IFN-γ and Ki-67, along with a marked reduction in PD-1 expression. A similar trend was observed in the TREM2^+^ + 1-MT group, showing upregulation of IFN-γ and Ki-67 and downregulation of PD-1. Notably, in the dual-treatment group (TREM2^+^ + CH223191 + 1-MT), IFN-γ and Ki-67 were further elevated, while PD-1 was further suppressed relative to either monotherapy group. To further assess the functional status of tumor-infiltrating CD8^+^ T cells, we performed multicolor flow cytometry gating on CD8^+^ populations (**Figures 6E, F**). The results indicated that the dual inhibition group had a significantly higher proportion of CD8^+^IFN-γ+ and CD8^+^Ki-67^+^ cells, accompanied by a substantial decrease in CD8^+^PD-1^+^ cells.

Together, these findings demonstrate that combined blockade of AHR and IDO1 synergistically enhances CD8^+^ T cell activation and proliferation while attenuating their exhaustion, thereby markedly restoring effector function within the TREM2^+^ tumor microenvironment.

### TREM2^+^ macrophages mediate CD8^+^ T cell immunosuppression via the IDO1/TDO2-Kyn-AHR pathway

**Figure 7:**
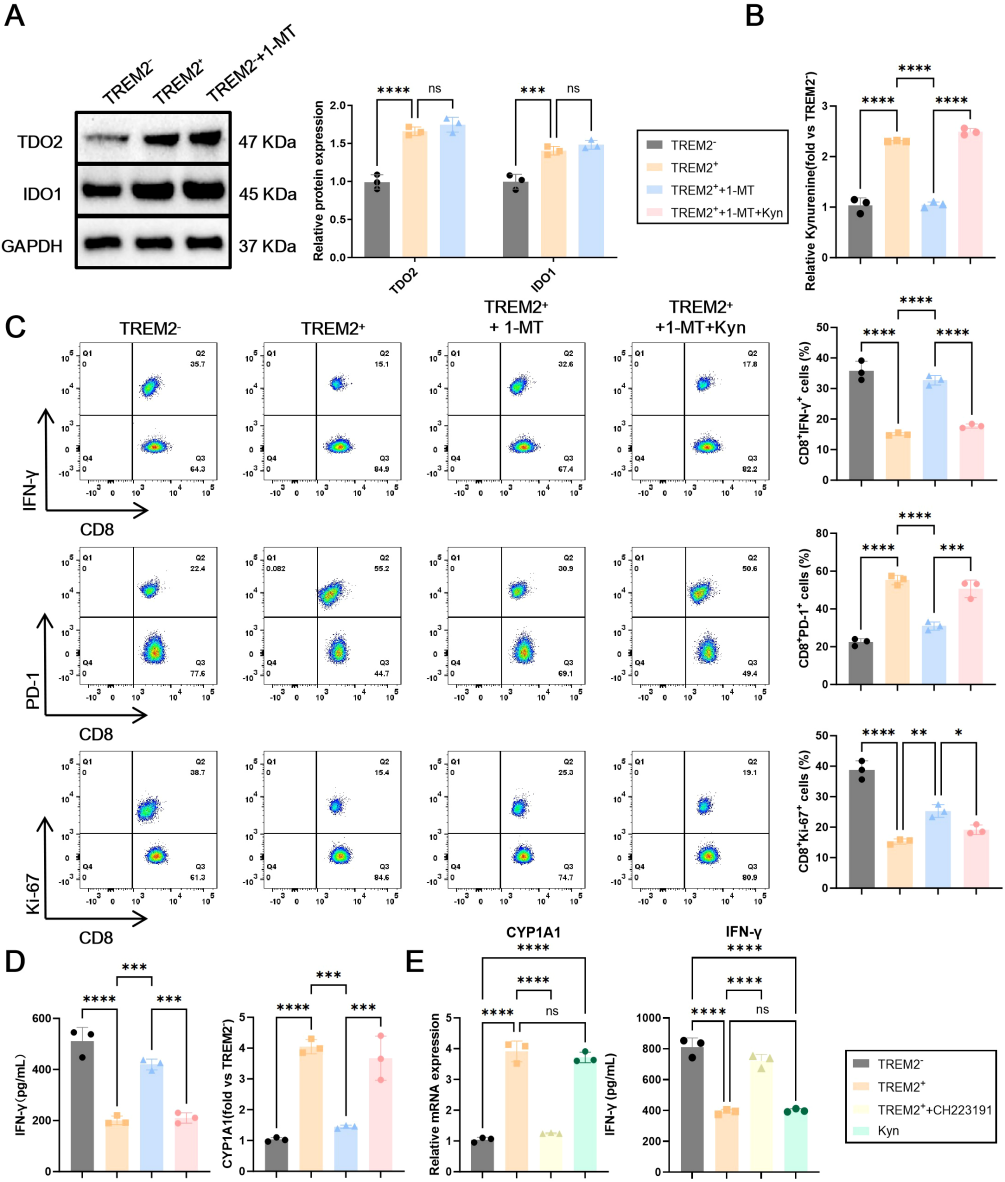
Functional and mechanistic role of Kyn in macrophage-mediated immunosuppression. **(A)** Western blot analysis of IDO1 and TDO2 protein levels; **(B)** Measurement of Kyn concentration in the supernatant using LC-MS/MS; **(C)** Flow cytometric analysis of CD8^+^ T cell expression of IFN-γ, PD-1, and Ki-67 under various treatment conditions, along with statistical comparisons; **(D)** Following exposure to conditioned media from different experimental conditions, CD8^+^ T cells were assessed for IFN-γ secretion (left) and CYP1A1 transcription levels (right); **(E)** RT-qPCR analysis of CYP1A1 expression (left) and ELISA-based quantification of IFN-γ levels in the culture supernatant (right). All cellular experiments were repeated three times. ns, *p* > 0.05; *p < 0.05; **p < 0.01; ***p < 0.001; ****p < 0.0001.

To determine whether TREM2+ macrophages produce Kyn through the IDO1/TDO2 pathway, we assessed the expression levels of metabolic enzymes and their associated metabolites. Both IDO1 and TDO2 protein levels were significantly elevated in TREM2+ macrophages relative to the TREM2- group, and treatment with 1-MT did not alter their expression (**Figure 7A**). Metabolite analysis further showed that Kyn levels in the supernatant of TREM2+ macrophages were markedly increased. Pre-treatment with 1-MT effectively reduced Kyn accumulation, whereas exogenous Kyn addition restored its concentration (**Figure 7B**). Together, these results suggest that TREM2+ macrophages catalyze tryptophan metabolism via the IDO1/TDO2 pathway, thereby increasing Kyn production.

To further verify whether the CD8+ T cell immunosuppressive effect induced by TREM2+ macrophages depends on Kyn, we evaluated T cell responses in conditioned medium (CM). Compared to the TREM2- CM control, CD8+ T cells treated with TREM2+ CM showed a significant reduction in IFN-γ+ cells, accompanied by increased PD-1+ and decreased Ki-67+ cell proportions, indicating a strong suppression of effector function and proliferation. Pre-treatment of TREM2+ macrophages with the IDO1 inhibitor 1-MT notably alleviated these suppressive effects. Reintroduction of exogenous Kyn under these conditions restored the inhibitory phenotype (**Figure 7C**). In the same experimental system, we analyzed CD8+ T cell functional markers and metabolic signaling pathways. Following TREM2+ CM exposure, IFN-γ production was significantly diminished, while transcription of CYP1A1—a canonical AHR target—was substantially increased. Inhibition of IDO1 activity by 1-MT partially restored IFN-γ secretion and reduced CYP1A1 expression. Conversely, exogenous Kyn supplementation once again reinforced the suppressive effect (**Figure 7D**). Collectively, these findings indicate that Kyn is not only sufficient but also essential as a soluble mediator in TREM2+ macrophage-induced CD8+ T cell immunosuppression.

To determine whether the immunosuppressive effect mediated by TREM2^+^ macrophages depends on the activation of AHR within CD8^+^ T cells, we performed targeted inhibition by applying the AHR antagonist CH223191 specifically to the T cell compartment. Compared to the TREM2- CM control, TREM2^+^ CM markedly induced transcriptional upregulation of CYP1A1 in CD8^+^ T cells, accompanied by a significant reduction in IFN-γ secretion. Pre-treatment of CD8^+^ T cells with CH223191 restored CYP1A1 expression to near-baseline levels and significantly increased IFN-γ production. Additionally, exogenous supplementation with Kyn (20 μM) recapitulated the suppressive effects of TREM2^+^ CM, which were likewise abrogated by CH223191 when administered to CD8^+^ T cells (**Figure 7E**). These findings demonstrate that TREM2^+^ macrophage-induced CD8^+^ T cell immunosuppression is dependent on AHR activation within the T cells, with Kyn acting as a critical upstream signaling molecule.

This study provides systematic evidence that TREM2^+^ macrophages mediate CD8^+^ T cell suppression through Kyn generated via the IDO1/TDO2 metabolic axis. Kyn functions both as a sufficient trigger and a necessary mediator of immunosuppression, exerting its effects by activating the AHR signaling pathway in CD8^+^ T cells.

### TREM2^+^ macrophages promote TC progression through immunosuppressive remodeling

**Figure 8:**
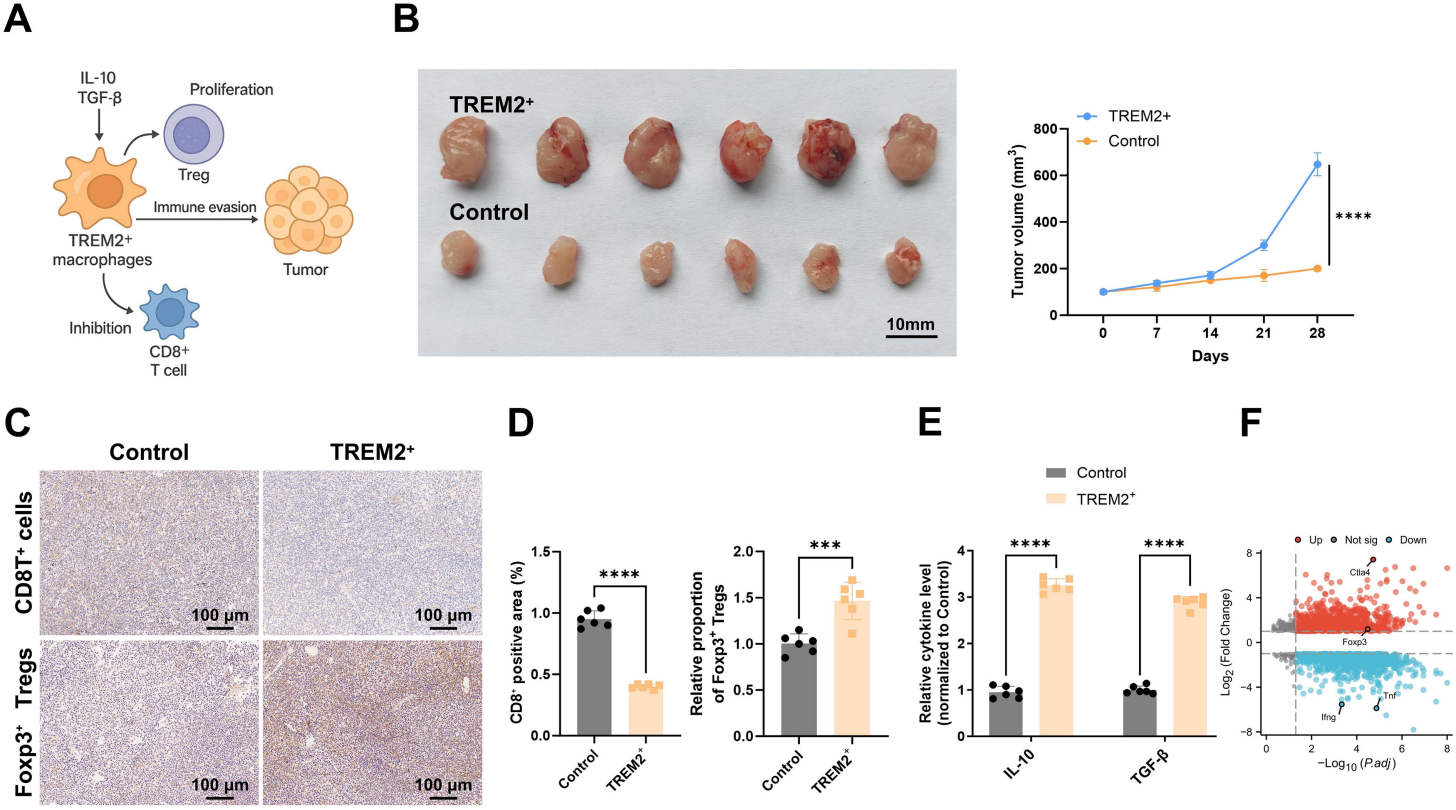
Pro-tumor function of TREM2^+^ macrophages and their immunoregulatory mechanism. **(A)** Schematic illustration of the mechanism by which TREM2^+^ macrophages facilitate immune evasion by secreting IL-10 and TGF-β to promote Treg proliferation, inhibit CD8^+^ T cell infiltration and effector function, thereby driving tumor immune escape; **(B)** Tumor size and growth curves in xenograft models comparing the TREM2^+^ macrophage-treated group and control group (n = 6); **(C)** Immunohistochemical staining of tumor tissues to evaluate the distribution and proportion of CD8^+^ T cells and Foxp3+ Tregs; brown staining indicates positive cells, bar = 100 μm; **(D)** Quantification of CD8^+^ T cell-positive area and Foxp3+ Treg cell proportion in tumor tissues by IHC; **(E)** ELISA analysis of the relative concentrations of the immunosuppressive cytokines IL-10 and TGF-β in tumor tissues; **(F)** Volcano plot illustrating differential expression of genes related to immunosuppression and effector T cell function. Each animal group included six mice. ns, *p* > 0.05; ***p < 0.001; ****p < 0.0001.

To elucidate the role of TREM2^+^ macrophages in TC progression, we further investigated their regulatory effects on the tumor immune microenvironment (**Figure 8A**). Tumor growth was first assessed in an *in vivo* xenograft model. Compared with the control group, mice receiving TREM2^+^ macrophage treatment exhibited accelerated tumor progression, with tumor volume showing sustained and significant increases throughout the observation period, indicating a pronounced pro-tumorigenic effect of TREM2^+^ macrophages (**Figure 8B**). IHC and ELISA were subsequently performed on xenograft thyroid tumor tissues. Immunohistochemical analysis showed that TREM2^+^ treatment reduced CD8^+^ T cell infiltration while increasing the proportion of Foxp3+ Tregs, suggesting the establishment of an immunosuppressive cellular landscape (**Figures 8C, D**). In parallel, ELISA quantification of tumor lysates showed significantly elevated levels of the immunosuppressive cytokines IL-10 and TGF-β following TREM2^+^ macrophage treatment, further supporting the notion that these cells promote immunosuppressive conditions via soluble mediators (**Figure 8E**).

To further delineate the immunosuppressive mechanisms and molecular basis of TREM2^+^ macrophage function, transcriptomic sequencing and differential gene expression analysis were conducted on tumor tissues from the TREM2^+^ and TREM2- groups. The results showed marked upregulation of immunosuppressive transcription factors and receptors, including Foxp3, CTLA-4, and PD-L1, in the TREM2^+^ group, accompanied by downregulation of effector T cell-associated genes such as Tnf and Ifng, indicating a profound disruption of immune equilibrium mediated by TREM2^+^ macrophages (**Figure 8F**).

To further validate these findings at the histological level, we performed additional analyses using residual tumor tissues collected from the *in vivo* experiments. As shown in **Supplementary Figure 4**, hematoxylin and eosin staining revealed enlarged necrotic areas and increased cellular density in tumors from the TREM2^+^ group (**Supplementary Figure 4A**). Ki67 immunohistochemistry showed a significantly higher tumor-cell proliferation index in the TREM2^+^ group (**Supplementary Figure 4B**). Assessment of macrophage polarization markers demonstrated an increased proportion of CD206-positive M2-like macrophages and a reduced proportion of CD86-positive M1-like macrophages in the TREM2^+^ group (**Supplementary Figure 4C**). CD8 and Granzyme B double immunofluorescence further confirmed a reduced proportion of functional CD8^+^ T cells in the TREM2^+^ group (**Supplementary Figure 4D**).

Collectively, these findings demonstrate that TREM2^+^ macrophages remodel the TC microenvironment by suppressing CD8^+^ T cell activity, increasing Treg infiltration, and secreting immunosuppressive cytokines, thereby facilitating tumor initiation and progression on multiple fronts.

### AHR binds the IDO1 promoter and drives its transcriptional activation

**Figure 9:**
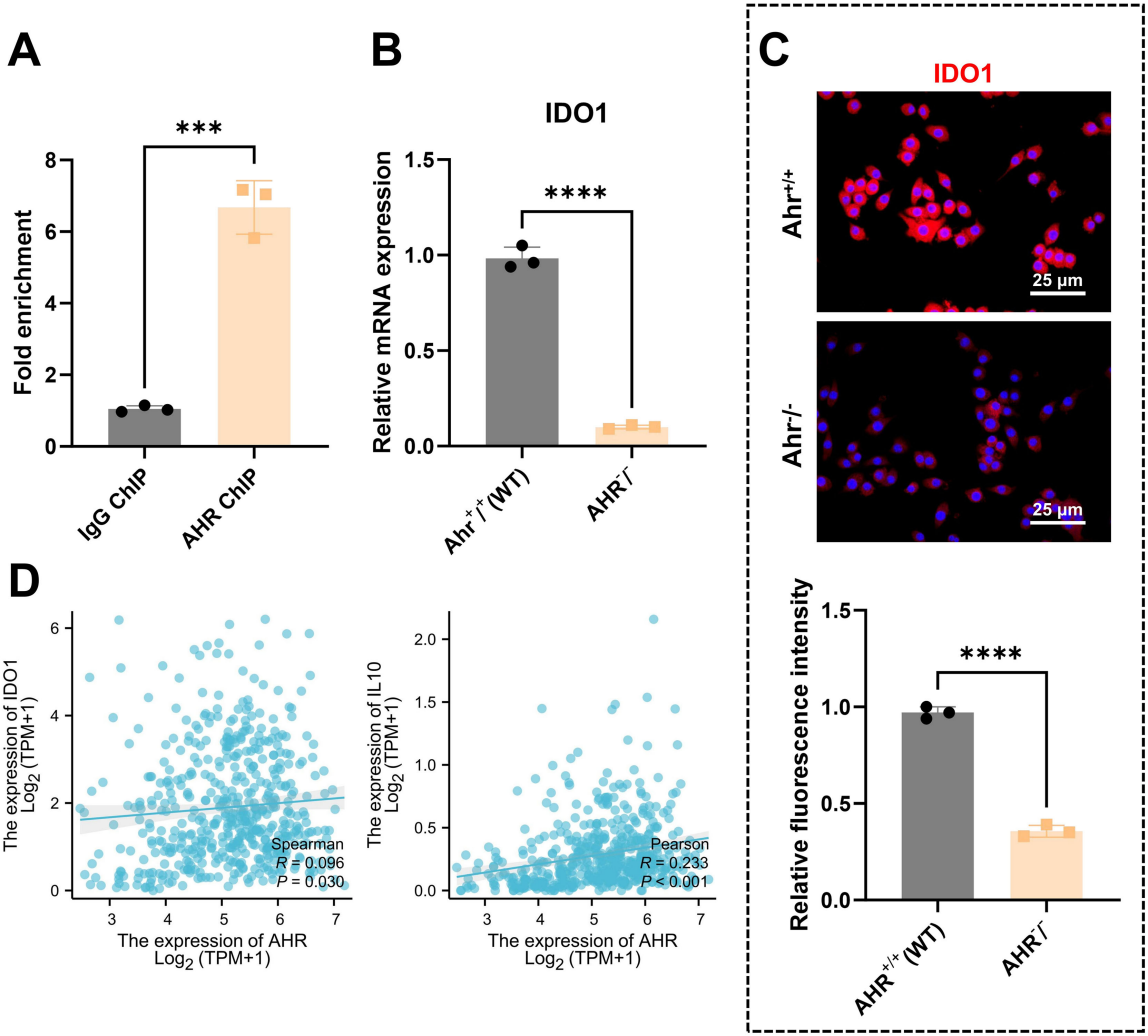
AHR regulates IDO1 transcription and the expression of immunosuppression-related genes. **(A)** ChIP-qPCR analysis of AHR antibody versus IgG control demonstrates differential enrichment at the IDO1 promoter region; **(B)** RT-qPCR quantification of IDO1 mRNA expression in Ahr+/+ and Ahr-/- macrophages; **(C)** IF staining of IDO1 protein in Ahr+/+ and Ahr-/- macrophages, with quantification of relative fluorescence intensity; bar = 25 μm; **(D)** Correlation analysis of AHR expression with IL10 and IDO1 levels in the TCGA-THCA cohort. Cell-based experiments were carried out in triplicate. ns, *p* > 0.05; ***p < 0.001; ****p < 0.0001.

To elucidate the direct regulatory mechanism by which AHR modulates IDO1 expression in macrophages, we performed ChIP-qPCR to assess AHR binding to the Ido1 promoter region. The results identified significant enrichment of AHR at the IDO1 promoter relative to the negative control, indicating a specific binding site for this transcription factor (**Figure 9A**).

To verify the functional role of AHR in regulating IDO1 transcription, we evaluated IDO1 mRNA and protein levels in AHR-knockout (AHR-/-) macrophages. RT-qPCR analysis indicated that IDO1 transcript levels were markedly reduced in AHR-/- cells relative to wild-type (AHR+/+) controls, which suggests that loss of AHR substantially impairs IDO1 expression (**Figure 9B**). IF co-staining further verified a corresponding decrease in IDO1 protein levels, with significantly diminished intracellular fluorescence signals (**Figure 9C**). To further investigate AHR’s role in regulating immunosuppressive genes, we conducted correlation analyses using TCGA-THCA data (https://portal.gdc.cancer.gov) between AHR and the expression of IDO1 and IL10. AHR expression positively correlated with both IL10 (Pearson r = 0.233, *p* < 0.001) and IDO1 (Spearman r = 0.096, *p* = 0.030), both of which are key immunosuppressive markers (**Figure 9D**).

Together, these findings demonstrate that AHR directly regulates IDO1 transcription and activates broader immunosuppressive signaling pathways, highlighting its pivotal role in macrophage functional reprogramming.

### Kyn enhances immune tolerance in the TC microenvironment via the TAM-Treg axis

**Figure 10:**
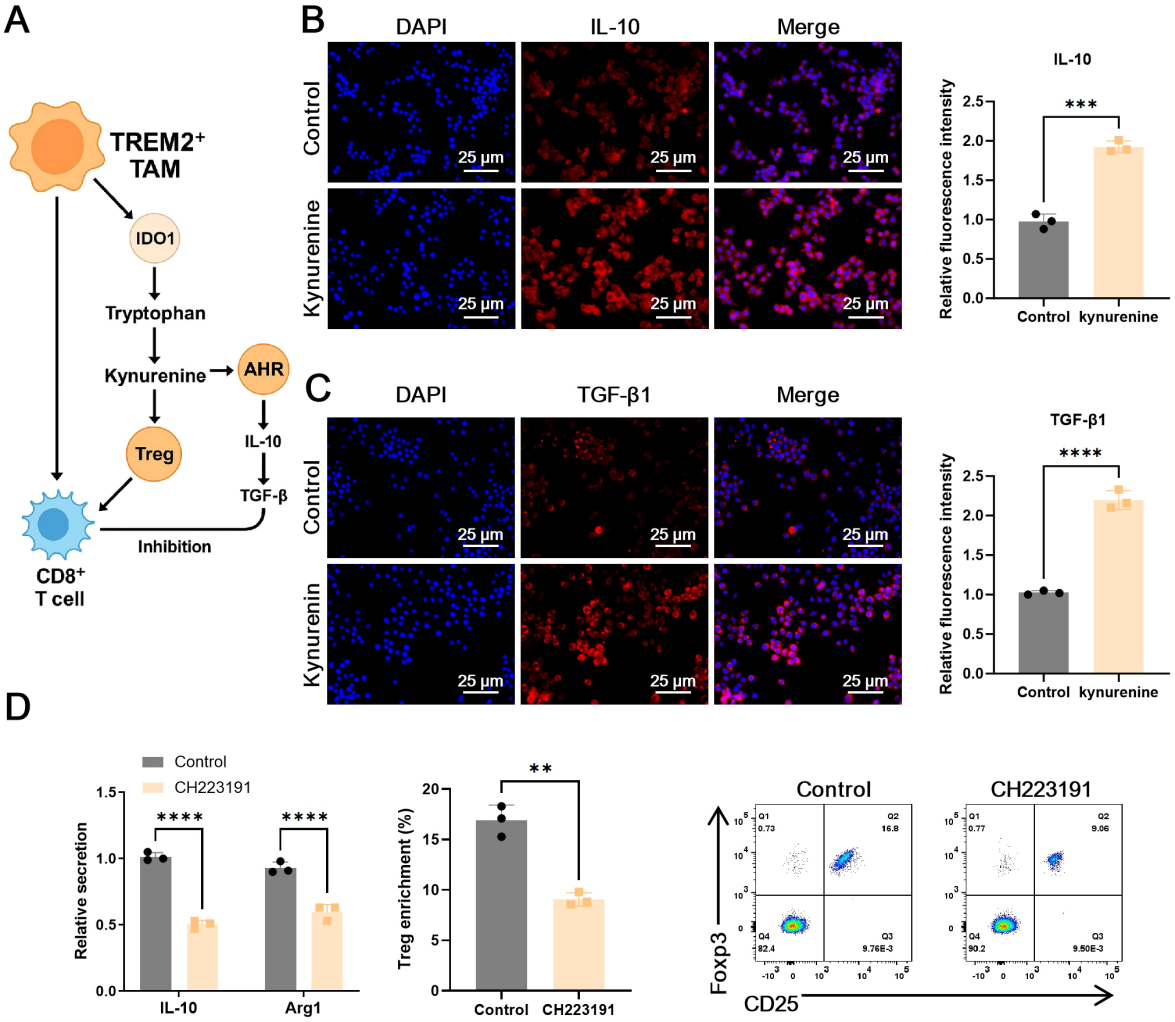
Kyn regulates immune tolerance in the TC microenvironment via the TAM-Treg axis. **(A)** Schematic illustration of the immunosuppressive mechanism mediated by the Kyn-AHR axis through the TAM-Treg pathway; **(B, C)** IF staining showing changes in IL-10 and TGF-β1 expression in Treg cells following exogenous Kyn treatment. Nuclei were counterstained with DAPI (blue), and target proteins were visualized in red; fluorescence intensity quantification is shown on the right. Bar = 25 μm; **(D)** Macrophages were treated *in vitro* with the selective AHR inhibitor CH-223191 (10 μM), then ELISA analysis of IL-10 and Arg1 secretion and flow cytometry quantification of Treg (CD4^+^CD25^+^Foxp3+) proportions in co-culture. All *in vitro* experiments were repeated three times. ns, *p* > 0.05; **p < 0.01; ***p < 0.001; ****p < 0.0001.

We hypothesized that TREM2+ TAMs promote the conversion of tryptophan into Kyn through high IDO1 expression, releasing Kyn into the tumor microenvironment, where it activates the AHR pathway within Treg cells. This activation subsequently promotes secreting IL-10 and TGF-β while suppressing CD8+ T cell effector function (**Figure 10A**). Functional assays were conducted to validate the regulatory effects of Kyn on Treg cells. Upon exposure to elevated levels of Kyn, Treg cells exhibited significantly increased secretion of IL-10 and TGF-β1, which indicates that Kyn enhances their immunosuppressive capacity (**Figures 10B, C**).

To further confirm the role of the Kyn-AHR signaling axis in TAM-mediated immunoregulation, *in vitro*-cultured macrophages were treated with the selective AHR inhibitor CH-223191 (10 μM) to block AHR signaling. Following AHR inhibition, levels of IL-10 and Arg1 secreted by TAMs were markedly reduced. Additionally, in a macrophage-T cell co-culture system, the proportion of Treg cells (CD4^+^CD25^+^Foxp3+) was significantly decreased, demonstrating that the Kyn-AHR axis plays a central role in the TAM-Treg immunoregulatory circuit (**Figure 10D**).

Collectively, these findings reveal that Kyn shapes the immunosuppressive microenvironment in TC via the TAM-Treg axis, with AHR signaling serving as a pivotal regulatory mediator.

### Combined targeting of AHR/IDO1 reverses immunosuppression in TC by reprogramming TAM polarization

**Figure 11:**
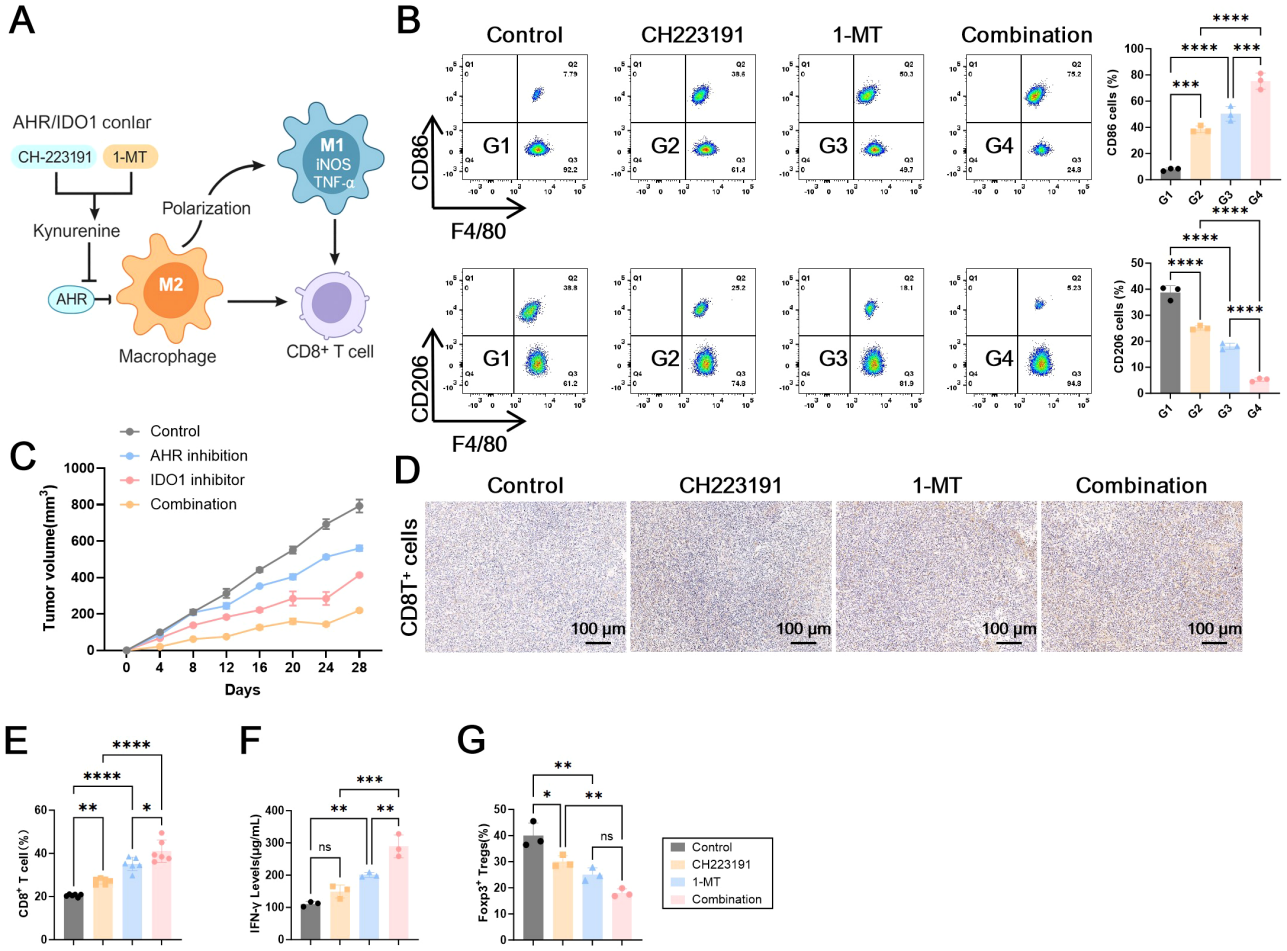
Combined targeting of AHR/IDO1 remodels TAM polarization and improves the immune microenvironment in TC. **(A)** Schematic diagram depicting the mechanism by which AHR/IDO1 signaling regulates TAM polarization and modulates CD8^+^ T cell activity; **(B)** Flow cytometry analysis of CD86^+^ (M1) and CD206^+^ (M2) macrophage populations under different treatment conditions (Control, AHR inhibitor, IDO1 inhibitor, Combination), with corresponding quantification; **(C)** Tumor volume measurements in murine TC models following the indicated treatments; **(D)** Immunohistochemical staining for CD8^+^ T cell infiltration in tumor tissues, with quantitative analysis shown in **(E)**; **(F)** ELISA analysis of IFN-γ levels across treatment groups; **(G)** Flow cytometry assessment of Foxp3+ Treg proportions in tumor tissues. ns, *p* > 0.05; *p < 0.05; **p < 0.01; ***p < 0.001; ****p < 0.0001.

To evaluate the therapeutic potential of targeting the Kyn-AHR immunometabolic axis, a combinatorial intervention was designed using the AHR antagonist CH223191 and the IDO1 inhibitor 1-MT. The effects of this regimen on TAM polarization and the immune microenvironment of TC were systematically assessed (**Figure 11A**).

Flow cytometry verified a significant phenotypic shift in TAMs. Compared with the control group, combined treatment led to a marked increase in CD86+ (M1-like) macrophages and a notable decrease in CD206+ (M2-like) populations among F4/80+ cells, which indicates that inhibition of the Kyn-AHR axis promotes TAM repolarization toward a pro-inflammatory M1 phenotype (**Figure 11B**).

In a BRAF-mutant TC mouse model, the combined intervention exhibited robust antitumor effects. Tumor volume was continuously monitored, and significant growth suppression was observed in the treatment group. By day 28, the combination therapy showed superior tumor inhibition relative to either monotherapy alone (**Figure 11C**).

Histological analyses further showed the impact of treatment on immune cell composition. Immunohistochemical staining identified a substantial increase in CD8+ T cell infiltration within tumor tissues following treatment (**Figures 11D, E**). ELISA results also showed a significant elevation of IFN-γ levels in the treatment group, suggesting enhanced antitumor immune activity and restoration of CD8+ T cell function mediated by dual inhibition of AHR and IDO1, thereby reversing the immunosuppressive microenvironment in TC (**Figure 11F**). Correspondingly, flow cytometric quantification indicated a significant reduction in immunosuppressive Treg (Foxp3+) cells after combined AHR/IDO1 inhibition (**Figure 11G**).

In summary, these findings demonstrate that combinatorial targeting of the Kyn-AHR immunometabolic axis effectively reprograms TAM polarization and enhances CD8^+^ T cell function, thereby reversing the immunosuppressive microenvironment in TC and significantly augmenting antitumor immunity.

### Critical role of TREM2^+^ macrophages in mediating immune tolerance via the AHR-IDO1-Kyn axis in TC

**Figure 12:**
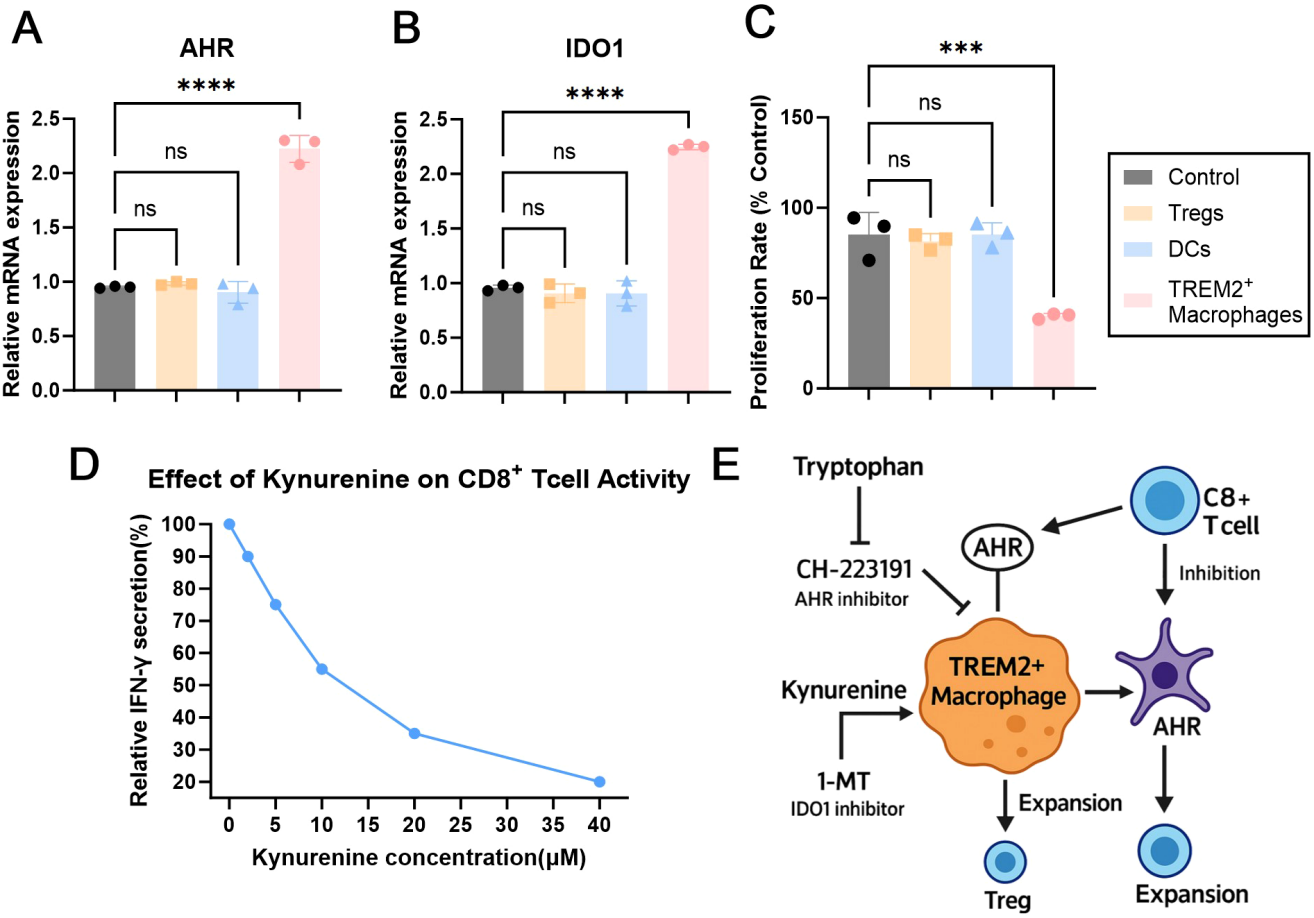
Dominant role of TREM2^+^ macrophages in the AHR-IDO1-Kyn immunometabolic axis. **(A, B)** RT-qPCR analysis of AHR and IDO1 gene expression levels in different immune cell populations (Control, Tregs, dendritic cells, and TREM2^+^ macrophages); **(C)** T cells were co-cultured with the indicated immune cell populations, and proliferation rates were assessed using CFSE dilution assays; **(D)** fitted curve illustrating the relationship between CD8^+^ T cell functional activity and Kyn concentration; **(E)** schematic illustration of the functional role of TREM2^+^ macrophages in the tryptophan-Kyn metabolic pathway. All *in vitro* experiments were repeated three times. ns, p > 0.05; ***p < 0.001; ****p < 0.0001. * indicates statistical significance between groups; ns, > 0.05; ***p < 0.001.

To further elucidate the molecular mechanisms by which TREM2^+^ macrophages contribute to immunosuppression, we performed a systematic analysis integrating gene expression profiling and functional assays (**Figure 12E**). RT-qPCR results indicated that AHR and IDO1 expression levels were significantly higher in TREM2^+^ macrophages than in control cells, Tregs, and dendritic cells, indicating cell type-specific upregulation of these genes in TREM2^+^ macrophages (**Figures 12A, B**). Co-culture experiments of T cells with different immune cell populations showed that TREM2^+^ macrophages markedly suppressed T cell proliferation, whereas control cells, Tregs, and dendritic cells had limited effects (**Figure 12C**). Moreover, a fitted curve illustrating the relationship between IFN-γ secretion by CD8^+^ T cells and Kyn concentration showed a clear dose-dependent decline in T cell activity as Kyn levels increased (**Figure 12D**).

Collectively, these findings—spanning gene expression, functional suppression, and metabolic dependence—demonstrate that TREM2^+^ macrophages play a central role in driving immunosuppression through the IDO1-Kyn-AHR axis.

### Activation of the IDO1-AHR axis promotes M2 polarization and shapes an immunosuppressive TC microenvironment

**Figure 13:**
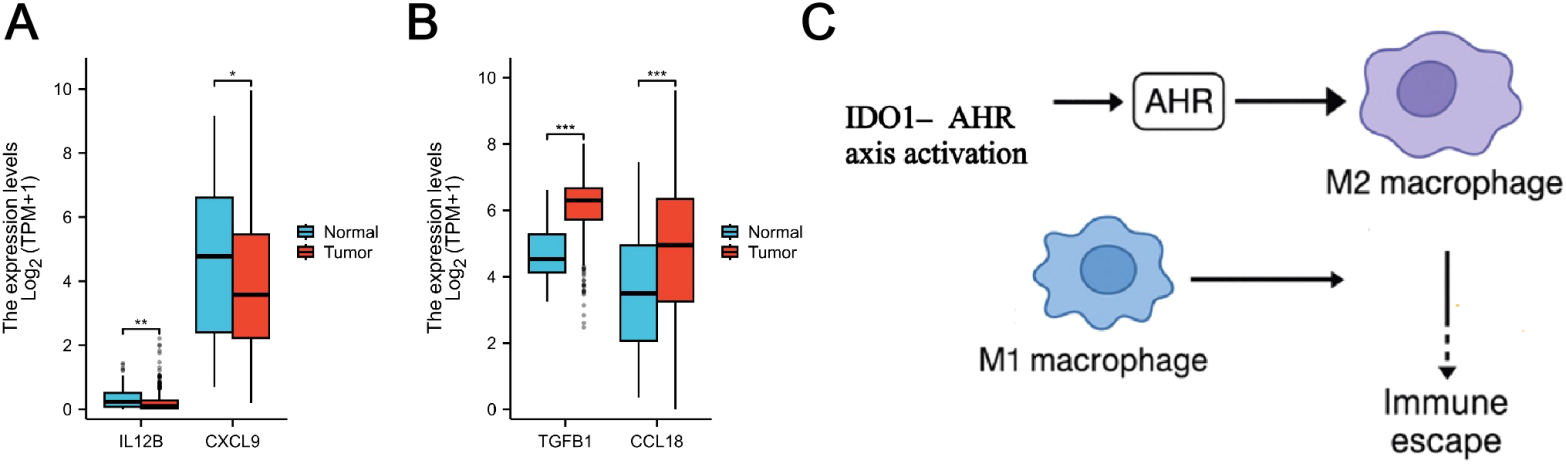
The IDO1-AHR pathway promotes M2 polarization of TAMs. **(A, B)** Based on TCGA-THCA data, gene expression levels of M1-associated markers (IL12B and CXCL9) and M2-associated markers (TGFB1 and CCL18) were compared between TC tumor tissues (n = 511) and normal tissues (n = 58), presented as Log2(TPM + 1); **(C)** mechanistic diagram showing how activation of the IDO1-AHR signaling axis drives the polarization of M1 macrophages toward an M2 phenotype, thereby contributing to the formation of an immune-evasive microenvironment. *p < 0.05; **p < 0.01; ***p < 0.001.

To further investigate how aberrant expression of genes related to the IDO1-AHR axis affects the immune microenvironment in TC (**Figure 13C**), we analyzed transcriptomic data from the TCGA database. The results showed that the expression of M1 macrophage-associated markers IL12B and CXCL9 was significantly lower in tumor tissues than in normal counterparts (**Figure 13A**), indicating suppression of pro-inflammatory and antitumor functions. In contrast, expression of M2 macrophage-associated markers TGFB1 and CCL18 was markedly elevated in tumor tissues (**Figure 13B**), suggesting a shift toward an immunosuppressive, tumor-promoting phenotype within the immune microenvironment.

Taken together, these findings indicate that activation of the IDO1-AHR pathway may drive the polarization from M1 to M2 macrophages, thereby fostering an immunosuppressive microenvironment that facilitates immune evasion.

## Discussion

This study systematically elucidated the functional and metabolic properties of TAMs within the TC immune microenvironment. For the first time, it identified a macrophage subset marked by TREM2 as a pivotal driver of tumor progression and immune evasion. By integrating high-dimensional single-cell technologies, including scRNA-seq and CyTOF, we captured a distinct TREM2^+^ TAM population characterized by unique immunological and metabolic signatures within the TC microenvironment. Further analyses using scATAC-seq, RT-qPCR, and ChIP-qPCR, combined with pharmacological inhibition and functional perturbation assays, showed that this subset significantly suppressed CD8^+^ effector T cell activity by activating the AHR-IDO1-Kyn metabolic axis, thereby promoting immunosuppressive remodeling. These findings fill a critical gap in our understanding of TREM2^+^ TAMs in TC (26) and provide a strong rationale for targeting TREM2^+^ TAMs and their associated metabolic pathways. Notably, dual blockade of AHR and IDO1 showed, in both *in vivo* and *in vitro* models, the ability to reprogram TAM polarization and restore antitumor immunity, underscoring the translational potential of targeting the “metabolism-immunity” interface.

In comparison with previous reports of TREM2^+^ macrophages in other solid tumors (9, 27, 28), this study expands the understanding of TREM2^+^ TAMs by uncovering a TC-specific mechanism of metabolic and immunological regulation. It demonstrates that macrophages within the tumor microenvironment are not only functionally distinct but also exhibit context-dependent modes of action. Unlike in other cancers, where immune suppression is primarily mediated through gene regulatory pathways, our data show that in PTC, TREM2^+^ TAMs suppress CD8^+^ T cell activity through an IDO1-driven Kyn-AHR metabolic axis, thereby shaping an immunosuppressive niche. This highlights the critical role of tumor-specific metabolic subenvironments and suggests that cancer-type-tailored interventions targeting TREM2-related pathways may significantly enhance the efficacy of immunotherapy. Together with *in vivo* validation in murine models, these results establish a coherent “mechanism-function-intervention” evidence chain.

Through AHR signaling analysis, we further verified that TREM2+ TAMs exhibit markedly enhanced Kyn metabolic activity within the tryptophan pathway. This metabolic feature is particularly prominent in the TC microenvironment and aligns with previously reported associations between immunosuppressive cytokine axes and effector T cell exhaustion (29). Integrated single-cell multi-omics analysis showed that TREM2+ TAMs, via Kyn-mediated AHR signaling, induced the expression of multiple downstream immune regulatory molecules, thereby establishing a profoundly immunosuppressive microenvironment. Experimental results showed that dual blockade of IDO1 and AHR signaling effectively reduced Kyn levels and significantly restored CD8+ T cell activity. These findings provide crucial experimental evidence supporting the feasibility of targeting metabolic pathways in immunotherapy and underscore the potential of precise metabolic regulation as a key strategy in future cancer immunotherapeutic approaches.

In terms of methodology and evidence hierarchy, a major strength of this study lies in its closed-loop design integrating single-cell multi-omics (scRNA-seq, scATAC-seq), spatial/histological validation, and functional/pharmacological interventions. This comprehensive approach systematically characterized the phenotype, metabolic profile, and signaling regulation of TREM2^+^ TAMs. Unlike previous studies that merely inferred chromatin accessibility, this work further incorporated ChIP-qPCR to demonstrate AHR binding to the IDO1 promoter directly and confirmed the functional signaling circuit in both *in vivo* and *in vitro* models, thus establishing a more complete and reproducible “mechanism-readout-intervention” evidence chain.

Overall, this study, through a single-cell multi-omics perspective, advances our understanding of the regulatory networks within the immune microenvironment of TC, particularly highlighting the central role of the AHR-IDO1-Kyn axis in TREM2^+^ TAM-mediated immunosuppression and tumor progression. These discoveries provide novel insights for TC immunotherapy, especially regarding strategies targeting the interplay between metabolism and immunity. However, the study also has certain limitations. As most analyses were conducted in murine models, further validation using human TC samples is necessary to confirm their applicability to human disease, and the heterogeneity of TREM2^+^ TAM subpopulations in human PTC requires larger single-cell cohorts for further resolution. The long-term efficacy and potential toxicity of the combination therapy also remain to be explored. In addition, some assessments of T-cell proliferative status used total tumor tissue lysates for immunoblotting. Because these samples contain both tumor cells and infiltrating immune cells, part of the observed Ki-67 signal may reflect intrinsic tumor-cell proliferation. Nevertheless, flow cytometric cell-specific analysis of the CD8^+^ T cell compartment provided independent evidence supporting restoration of T-cell proliferative capacity and further strengthened the immunostimulatory effect of the combined intervention. Future research should verify these findings across broader clinical contexts and develop new combinatorial immunotherapeutic strategies that directly target Kyn metabolism and TAM function, thereby enhancing the precision and efficacy of TC treatment and ultimately improving patient outcomes.

## Data Availability

The original contributions presented in the study are included in the article/**Supplementary Material**. Further inquiries can be directed to the corresponding author.

## Supplementary material

Supplementary Figure 1 Workflow of multi-omics integration including transcriptomics, chromatin accessibility, and cell-cell communication networks. This figure illustrates the multi-omics analytical framework employed in the present study, which integrates transcriptomic sequencing, scRNA-seq, and chromatin accessibility profiling. These datasets were analyzed collectively and combined with inferred intercellular communication networks to construct a comprehensive immune regulatory map.

Supplementary Figure 2 *In vivo* validation workflow of TREM2^+^ macrophages in a TC xenograft model. This figure outlines the *in vivo* experimental procedure. BMDMs were isolated from mice, differentiated with GM-CSF, and subsequently treated with IL-4 and TGF-β to induce TREM2high expression. TREM2^+^ macrophages were then purified via flow cytometric sorting and co-cultured with isolated CD8^+^ T cells. Functional assessments included cytokine secretion (ELISA), activation/exhaustion markers (flow cytometry), Kyn levels (LC-MS/MS), and signaling pathway proteins (Western blot). Pharmacological interventions with AHR and IDO1 inhibitors were also applied to evaluate their roles in immune regulation.

Supplementary Figure 3 *In vitro* functional validation workflow of TREM2^+^ macrophages. This figure presents the *in vitro* experimental workflow. BMDMs were isolated from murine bone marrow, induced to differentiate with GM-CSF, and further stimulated with IL-4 and TGF-β to achieve TREM2high expression. TREM2^+^ macrophages were sorted by flow cytometry and co-cultured with purified CD8^+^ T cells. Functional analyses included cytokine secretion (ELISA), activation/exhaustion marker profiling (flow cytometry), Kyn quantification (LC-MS/MS), and assessment of signaling proteins (Western blot). Concurrent AHR and IDO1 inhibition served to investigate their involvement in immune modulation.

Supplementary Figure 4 Supplementary histological and immunological characterization of tumor tissues from the TREM2^+^ and TREM2- groups. **(A)** Hematoxylin and eosin (HE) staining; **(B)** Ki67 immunohistochemical staining and quantitative analysis; **(C)** immunohistochemical staining and quantitative analysis of M1 (CD86) and M2 (CD206) macrophages; **(D)** CD8 and Granzyme B double immunofluorescence staining and quantitative analysis. CD8 is shown in green, Granzyme B in red, and nuclei are counterstained with DAPI in blue. Scale bar = 20 μm. Each animal group included six mice. * indicates comparison between two groups; ****p* < 0.001.

## Glossary

AHR: Aryl Hydrocarbon Receptor
BMDMs: Bone Marrow-Derived Macrophages
BSA: Bovine Serum Albumin
Bulk RNA-seq: Bulk RNA Sequencing
CM: Conditioned Medium
ChIP-qPCR: Chromatin Immunoprecipitation–Quantitative Polymerase Chain Reaction
CyTOF: Cytometry by Time-of-Flight
DEG: Differentially Expressed Gene
FBS: Fetal Bovine Serum
FDR: False Discovery Rate
GEO: Gene Expression Omnibus
GM-CSF: Granulocyte-Macrophage Colony-Stimulating Factor
GO: Gene Ontology
GSEA: Gene Set Enrichment Analysis
ICIs: Immune Checkpoint Inhibitors
IDO1: Indoleamine 2,3-Dioxygenase 1
IF: Immunofluorescence
IHC: Immunohistochemistry
KEGG: Kyoto Encyclopedia of Genes and Genomes
Kyn: Kynurenine
PBS: Phosphate-Buffered Saline
PTC: Papillary Thyroid Carcinoma
qPCR: Quantitative PCR
ROC: Receiver Operating Characteristic
scATAC-seq: Single-Cell Assay for Transposase-Accessible Chromatin Using Sequencing
scRNA-seq: Single-Cell RNA Sequencing
ssGSEA: Single-Sample Gene Set Enrichment Analysis
TAMs: Tumor-Associated Macrophages
TC: Thyroid Cancer
Treg cells: Regulatory T Cells
UHPLC-MS/MS: Ultra-High-Performance Liquid Chromatography–Tandem Mass Spectrometry
1-MT: 1-Methyl-D-Tryptophan
