# Supplementary material for: Targeting the AHR–IDO1–kynurenine pathway in TREM2+ macrophages restores antitumor immunity in thyroid cancer
Source: Front Immunol. 2026 Jul 21;17:1826288. doi: 10.3389/fimmu.2026.1826288 (PMC13433865; doi:10.3389/fimmu.2026.1826288)
Supplement: Supplementary file 1 [file Table1.docx]

**Table S1. Protein Information.**

| **Protein** | **Supplier/Catalog Number** | **kDa** | **Dilution** |
| --- | --- | --- | --- |
| TREM2 | Abcam/ab209814 | 25 | 1:1,000 |
| PD-L1 | Abcam/ab205921 | 40 | 1:1,000 |
| MHC-II | Abcam/ab139365 | 33 | 1:1,000 |
| t-STAT1 | Abcam/ab281999 | 91 | 1:1,000 |
| p-STAT1 | Abcam/ab278713 | 91 | 1:1,000 |
| t-STAT5 | Abcam/ab230670 | 90 | 1:1,000 |
| p-STAT5 | Abcam/ab278764 | 90 | 1:1,000 |
| IFN-γ | Abcam/ab9657 | 17 | 1:1,000 |
| PD-1 | Abcam/ab52587 | 55 | 1:1,000 |
| Ki-67 | Abcam/ab15580 | 359 | 1:1,000 |
| TDO2 | Abcam/ab121010 | 47 | 1:1,000 |
| IDO1 | Abcam/ab106134 | 45 | 1:1,000 |
| GAPDH | Abcam/ab8245 | 37 | 1:1,000 |

**Table S2. Antibody Information.**

| **Protein** | **Supplier/Catalog Number** | **Dilution** |
| --- | --- | --- |
| TREM2 | Abcam/ab209814 | 1:200 |
| CD8 | CST/85336 | 1:200 |
| Foxp3 | Abcam/ab20034 | 1:200 |
| AHR | CST/83200 | 1:200 |
| IDO1 | Abcam/ab106134 | 1:200 |
| IL-10 | Abcam/ab133575 | 1:100 |
| TGF-β1 | Thermo Fisher Scientific/MA5-23795 | 1:500 |

**Table S3. RT-qPCR Primer Sequences (Mouse).**

| **Gene** | **Primer Sequence (5′→3′)** |
| --- | --- |
| Trem2 | F: TCATCGAGTTTCGAGGGTGC  R: GCTGTCTTCCCCACTCAACA |
| PD-L1 | F: AGGTGCGTGATTTTGAGGGA  R: TATCTGCAGGCGAGGAGACT |
| MHC-II | F: AAGCTTTGACCCCCAAGGTG  R: GCTTGAGGAGCCTCATTGGT |
| Ido1 | F: GGGTCAGTGGAGTAGACAGC  R: GTCCAGGAACTGAGTTCCGT |
| Ahr | F: TTCAGAACTGACTCCACCGC  R: CCGGGTGTGATATCGGGAAG |
| Gapdh | F: GAAGGTCGGTGTGAACGGAT  R: ACTGTGCCGTTGAATTTGCC |
